# A New *In Vivo* Model to Study Protective Immunity to Zika Virus Infection in Mice With Intact Type I Interferon Signaling

**DOI:** 10.3389/fimmu.2018.00593

**Published:** 2018-03-22

**Authors:** Loulieta Nazerai, Amalie Skak Schøller, Peter Overbeck Sharma Rasmussen, Søren Buus, Anette Stryhn, Jan Pravsgaard Christensen, Allan Randrup Thomsen

**Affiliations:** Department of Immunology and Microbiology, University of Copenhagen, Copenhagen, Denmark

**Keywords:** Zika virus, mouse model, adaptive immunity, T cells, B cells, memory, protection

## Abstract

The association between recent Zika virus (ZIKV) infection and neurological complications, microcephaly in the fetus, and Guillain–Barré syndrome in adults underscores the necessity for a protective vaccine. Rational vaccine development requires an in-depth understanding of the mechanisms which could protect against infection with this virus. However, so far, such an analysis has been hampered by the absence of a suitable small animal model. Unlike the situation in humans, ZIKV only replicates effectively in the peripheral organs of mice, if type I IFN signaling is interrupted. As type I IFN also impacts the adaptive immune response, mice with such a defect are not optimal for a comprehensive immunological analysis. In this report, we show that even in wild-type (WT) mice i.c. infection with low doses of virus causes marked local virus replication and lethal encephalitis in naïve mice. Furthermore, peripheral infection of WT mice with low doses of virus induces a significant immune response, which provides long-lasting protection of WT mice from a fatal outcome of subsequent i.c. challenge. Therefore, combining peripheral priming with later i.c. challenge represents a new approach for studying the adaptive immune response to ZIKV in mice with an intact type I IFN response. In this study, we focused on the mechanisms underlying resistance to reinfection. Using a combination of adoptive transfer, antibody-based cell depletion, and gene targeting, we show that the key protective factor in type I IFN replete mice is humoral immunity. CD8 T cells are not essential in mice with preformed specific antibodies, but under conditions where initial antibody levels are low, effector CD8 T cells may play a role as a back-up system. These results have important implications for our understanding of natural immunity to ZIKV infection and for Zika vaccine design.

## Introduction

Zika virus (ZIKV) is one of the most recent viral pathogens to cause global public health concern. Nevertheless, ZIKV is not a newly identified virus since it was first isolated almost 70 years ago from a sentinel monkey in the Zika forest in Uganda, and it has since been assigned to the flaviviridae family of positive-stranded RNA viruses along with yellow fever, Dengue, West Nile, and Japanese Encephalitis virus (1). The first human case was reported in 1954, and by the end of the twentieth century, the virus was detected in a wide geographical area, including Thailand, Philippines, Vietnam, Indonesia, Malaysia, Pakistan, and India (2–4). In spite of its wide geographical distribution, the virus had maintained a relatively low profile until 2007 with only 14 documented cases of human disease attributed to ZIKV infection. Therefore, the ZIKV outbreak on the Micronesian island of Yap that year, which resulted in 70% of the population being infected, raised considerable concern (5). A larger outbreak followed in 2013 in French Polynesia, while in 2015, ZIKV emerged in the Americas for the first time and within a year had spread to over 50 countries (6, 7). Its fast paced global spread combined with accumulating evidence linking prenatal ZIKV infection to an increased rate of babies born with microcephaly and other neurodevelopmental abnormalities led the WHO to declare it a public health emergency of international concern (8, 9). In addition to neonatal microcephaly, ZIKV infection has been shown to affect also the adult population by attacking the peripheral nervous system and causing a neurodegenerative disease called Guillain–Barré syndrome in a few infected people (10, 11).

The ZIKV genome consists of a single-stranded, positive-sense RNA of approximately 11 kb in length and it encodes a single open reading frame. Translation of the viral RNA generates one polyprotein that is subsequently proteolytically processed into 10 mature proteins: 3 structural (C, prM/M, and E) and 7 non-structural (NS1, NS2A, NS2B, NS3, NS4A, NS4B, and NS5) (12). Cryo-electron microscopy studies have revealed a high similarity in virion organization and structure of ZIKV with other flaviviruses, especially Dengue virus types 3 and 4 (13–15). However, despite the similarity at the molecular level that ZIKV shares with other flaviviruses, it is distinct in its ability to cause transplacental infection, fetal abnormalities, and vector independent transmission through body fluids in humans (16, 17).

In humans, ZIKV suppresses host innate immune response by employing multiple strategies to block the induction of type-I IFN as well as downstream IFN-stimulated genes. The virus has been found to cause degradation of the antiviral transcriptional activator STAT-2 ultimately resulting in reduced type I/III IFN-mediated signaling (18, 19). The efficient evasion of type I IFN responses allows the virus to replicate to substantial titers and cause disease in humans. In mice, however, probably due to some species-specific immune evasion mechanisms, peripheral viral replication is limited and does not suffice to cause significant pathology. To enable its study, several murine models have been developed most of them with an absent or suppressed type I IFN signaling to allow viral replication (20–22). These models haven proven useful in characterizing certain aspects of ZIKV infection; however, there are intrinsic limitations as to what you can learn about the adaptive antiviral defense in biological systems where a critical component of the early host response is absent. Bearing that in mind, an animal model with an intact innate immune system is likely to prove advantageous in elucidating the complex interplay of the host’s immune system with ZIKV.

In this study, we have used two different strains of adult wild-type (WT) mice with an intact immune system to establish an *in vivo* model of ZIKV infection and characterize aspects of ZIKV protective immunity. For this purpose, we have utilized a number of gene-targeted mouse strains lacking critical components of the adaptive immune system and performed *in vivo* cell depletion as well as adoptive transfer assays, and we clearly document the dominant role of antibodies (Abs) in clinical protection but also a potential contribution of cell-mediated immunity.

## Materials and Methods

### Mice

Female BALB/c and C57BL/6 (B6) wild-type (WT) mice as well as β2-microglobulin-deficient (β_2_m^−/−^) and MHC class II-deficient (Aβ^−/−^) mice on a B6 background were obtained from Taconic farms and maintain under specific pathogen-free conditions. B cell-deficient mice (μMT/μMT, B6.129S2-Igh-6tm1Cgn/J), TCRβ-deficient mice (TCRβ^−/−^, B6.129P2-Tcrbtm1Mom/J), CD8-deficient mice (CD8^−/−^, B6.129S2-Cd8atmMak/J), CXCR5-deficient (CXCR5^−/−^, B6.129S2(Cg)-Cxcr5tm1Lipp/J) mice, and CD40L-deficient (CD40L^−/−^, B6.129S2-Cd40lgtm1lmx/J) mice were all obtained from the Jackson Laboratory (Bar Harbor, ME, USA). IFN-γ/perforin double-deficient (IFN-γ/Prf^−/−^) mice on a B6 background were produced as previously described (23) and maintained locally.

All mice used in this study were 7–10 weeks old and were housed under SPF conditions at the ALAAC accredited animal facility at the Panum Institute (Copenhagen, DK). Mice coming from outside sources were allowed to rest for at least 1 week before entering an experiment.

### Virus Preparation and Quantitation

Zika virus, strain MR766 (Uganda, 1947), was obtained from American Type Culture Collection (ATCC) (Manassas, VA, USA) and was propagated in Vero cells (ATCC CCL-81) grown in DMEM containing 10% FBS, glutamine, and antibiotics (penicillin and streptomycin). The titer of the virus stock was determined based on the number of plaque-forming units (pfu) in semi-confluent monolayers of Vero cells. Specifically, 10-fold serial dilutions of the virus stock were prepared and incubated for 2 h on Vero cell monolayers that were seeded a day earlier in 24-well plates. After the 2 h incubation, cells were overlayed with medium containing 0.9% methylcellulose and were further incubated for 5 days (37°C, 5% CO_2_). After fixation with 4% formaldehyde, cells were stained with 0.1% crystal violet for plaque visualization.

For quantitation of virus in the organs of mice, the organs were first homogenized in PBS to yield 10% suspensions and viral titers were subsequently determined as described above. The detection limit of the assay was 250 pfu/g of organ.

For the experiments regarding yellow fever virus (YFV), virus stock of strain YF-17D was produced and quantified as previously described (24).

### Immunization and i.c. Challenge

Unless stated otherwise, mice were immunized by i.v. injection of 1 × 10^3^ pfu ZIKV MR766 in 300 µl. During viral challenge, the mice were deeply anesthetized and, unless otherwise stated, 1 × 10^3^ pfu ZIKV MR766 in 30 µl was inoculated i.c. Health status and weight were monitored daily after i.c. challenge, and mice were euthanized when severe signs of illness along with a weight loss of or exceeding 25% of the initial weight were recorded. Immunization with YFV was done and mice were monitored as previously described (24).

### Titration of Neutralizing Ab

Neutralizing antibodies in the serum of mice immunized with ZIKV were determined by an *in vitro* plaque reduction assay. Twofold dilutions of the mouse sera were prepared using Vero cell media (DMEM 1965 NaHCO_3_ medium supplemented with penicillin and streptomycin, l-glutamine, Na-pyruvate, and 1% FBS) and incubated for 1 h (37°C, 5% CO_2_) with approximately 50 pfu ZIKV. The mixtures were subsequently transferred in duplicates to 24-well plates that had been seeded the day before with 1 × 10^5^ VERO cells per well. The plates were incubated for 2 h (37°C, 5% CO_2_) before the addition of the overlay medium and further incubated for 5 days. The plaques formed in each well were visualized and counted as previously described, and the neutralizing antibody titers were determined based on the highest serum dilution that neutralized more than 50% of the viral plaques.

### Flow Cytometry Analyses

Spleens were removed aseptically and transferred to Hanks Balanced Salt Solution (HBSS). Single-cell suspensions were obtained by pressing the spleens through a 70-µm nylon cell strainer, followed by centrifugation and two washes in HBSS. Approximately 2 × 10^6^ splenocytes were transferred to U-bottom 96-well microtiter plates and were incubated for 20 min (4°C in the dark) with PE-conjugated H-2D^b^ tetramers for ZIKV E_294–302_ (25) and subsequently stained for an additional 20 min (4°C in the dark) for relevant cell-surface markers. Cells to be stained for intracellular granzyme B were then permeabilized using the BD Biosciences FoxP3 staining protocol. Next, the cells were centrifuged, washed, fixed in 1% PFA, and finally resuspended in PBS and stored at 4°C until flow cytometric analysis. Cell samples were analyzed using FACS LSRFortessa cytometer (BD Biosciences), and the data were analyzed using FlowJo software version 10 (Tree Star).

### *In Vivo* CD4 and/or CD8 T-Cell Depletion

A combination of two monoclonal antibodies (YTS 169 and YTS 156) was used for *in vivo* depletion of CD8 T cells, while YTS191 was used for *in vivo* depletion of CD4 T cells; hybridomas producing these monoclonals were kindly provided by S. Cobbold (Sir Williams Dunn School of Pathology, Oxford, U.K.) (26). Mice to be depleted were injected i.p. with 200 µg of relevant antibody or antibody combination 1 day prior to i.c. challenge and with 100 µg of antibody 1 and 4 days post challenge. The efficiency of the cell depletion was confirmed by flow cytometric analysis of splenocytes on day 7 post i.c. challenge.

### Adoptive Transfer of Serum or Splenocytes

Serum was collected from mice immunized with 1 × 10^3^ pfu/300μl i.v. ZIKV 4 weeks earlier, and was transferred to naive recipient mice. Mice received 500 µl serum i.v. and 500 µl serum i.p. 3 days prior to i.c. challenge and 300 µl serum i.v. again 1 day before i.c. challenge. We have previously found that this regimen results in serum Ab levels about 1/3 of that in the donor serum (27, 28). Serum from naive mice was used as control.

Splenocytes were collected from similarly immunized mice and transferred to HBSS. Single-cell suspensions were obtained by pressing the spleens through a fine mesh (70 µm) followed by centrifugation and two washes in HBSS, after which cells were counted, and 7 × 10^7^ cells were injected i.v. into naive recipient mice 2 days prior to i.c. challenge. Splenocytes from naive mice or PBS were used as control.

### Statistical Evaluation

GraphPad Prism Software (version 7) was used for the statistical analysis. Quantitative results were compared using a nonparametric Mann–Whitney *U*-test and a *p*-value of <0.05 was considered evidence of a statistically significant difference.

## Results

### WT Immunocompetent Mice Are Susceptible to i.c. Infection With ZIKV

Peripheral inoculation of ZIKV does not cause readily detectable disease in WT mice. Given that a WT mouse model would be of great importance in studying ZIKV pathogenesis, and based on our experience from working with a mouse model for YFV (24), we wanted to investigate whether we could induce clinical disease by introducing ZIKV directly into the brain of WT mice.

**Figure 1:**
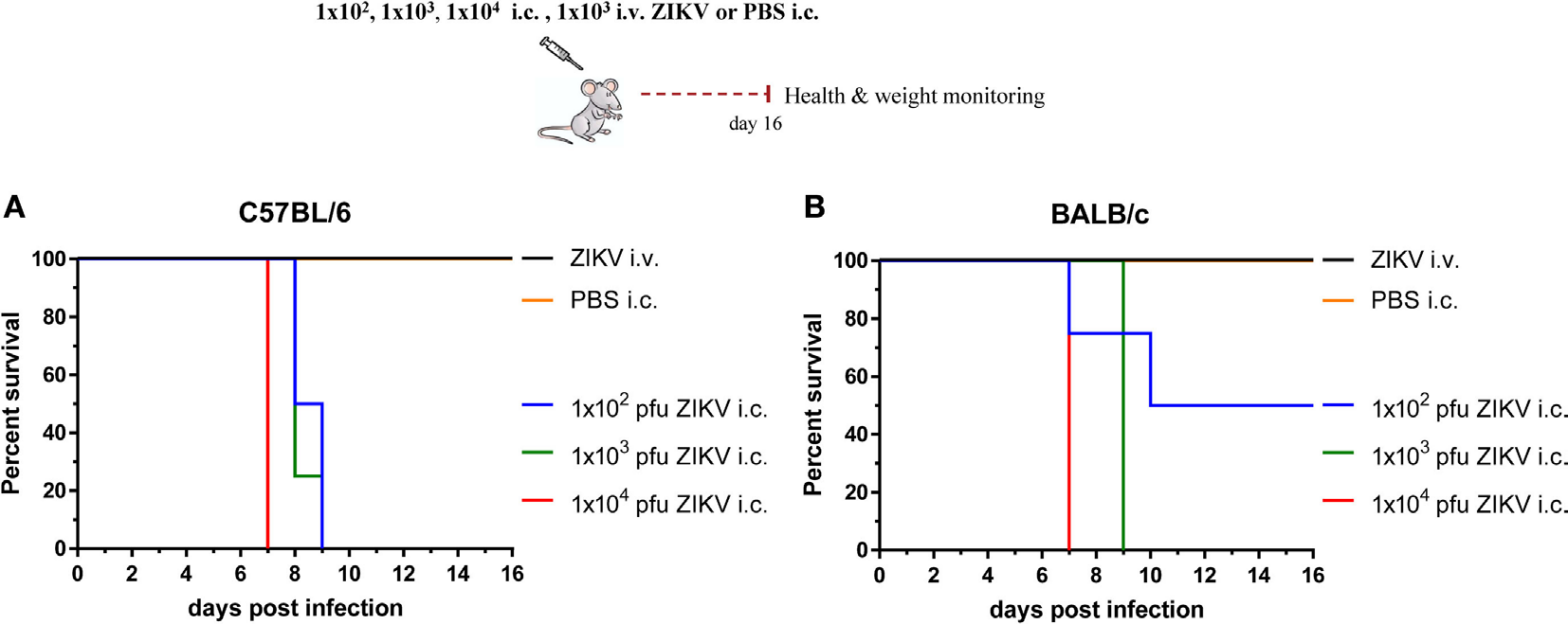
Determination of a suitable dose for i.c. challenge. Wild-type C57BL/6 **(A)** and BALB/c **(B)** mice were infected with 1 × 10^2^, 1 × 10^3^, or 1 × 10^4^ pfu Zika virus (ZIKV) i.c., and their weight, health, and survival were monitored daily for 16 days. Groups of mice injected with 1 × 10^3^ pfu ZIKV i.v. or PBS i.c. were included for comparison. A weight loss of 25% or more of initial body weight was used as humane end-point. *n* = 5 mice/group, *p < 0.05.

To that end, we challenged two different strains of WT mice (C57BL/6 and BALB/c) intracerebrally (i.c.) with 1 × 10^2^, 1 × 10^3^, or 1 × 10^4^ pfu of ZIKV and monitored their health and weight daily (data not shown). Unlike infection by the intravenous (i.v.) route, which did not lead to significant morbidity in any of the tested strains, i.c. infection induced almost the same pattern of severe disease in both mouse strains. In C57BL/6 mice, all three i.c. doses resulted in acute weight loss starting around day 4 post infection that progressed into severe disease by day 8 post infection, at which time point mice had lost more than 25% of their initial weight and had to be euthanized (Figure 1A). In BALB/c mice, the intermediate and high i.c. doses gave the same survival pattern as in C57BL/6, while some of the mice challenged with the lowest dose survived (Figure 1B). This suggests that, while minor differences may exist between the two mouse strains, the overall pattern was more or less the same.

In order to have a lethal challenge dose that would yield uniform pathogenesis in both strains, yet at the same time would not overwhelm the mice with high viral loads that might potentially impend the observation of small immunological differences, we chose to use the intermediate i.c. dose (1 × 10^3^ pfu) for the rest of our challenge experiments.

### Peripheral Inoculation With Low Doses of ZIKV Protects WT Mice Against Subsequent Lethal i.c. Infection With ZIKV

Consistent with findings from recent studies (22, 29), we did not observe any clinical signs of disease when we introduced the virus peripherally (Figure 1) and we were not able to detect production of infectious virus (<250 pfu/g organ) neither in blood nor in a number of organs tested (spleen, liver, and kidney).

**Figure 2:**
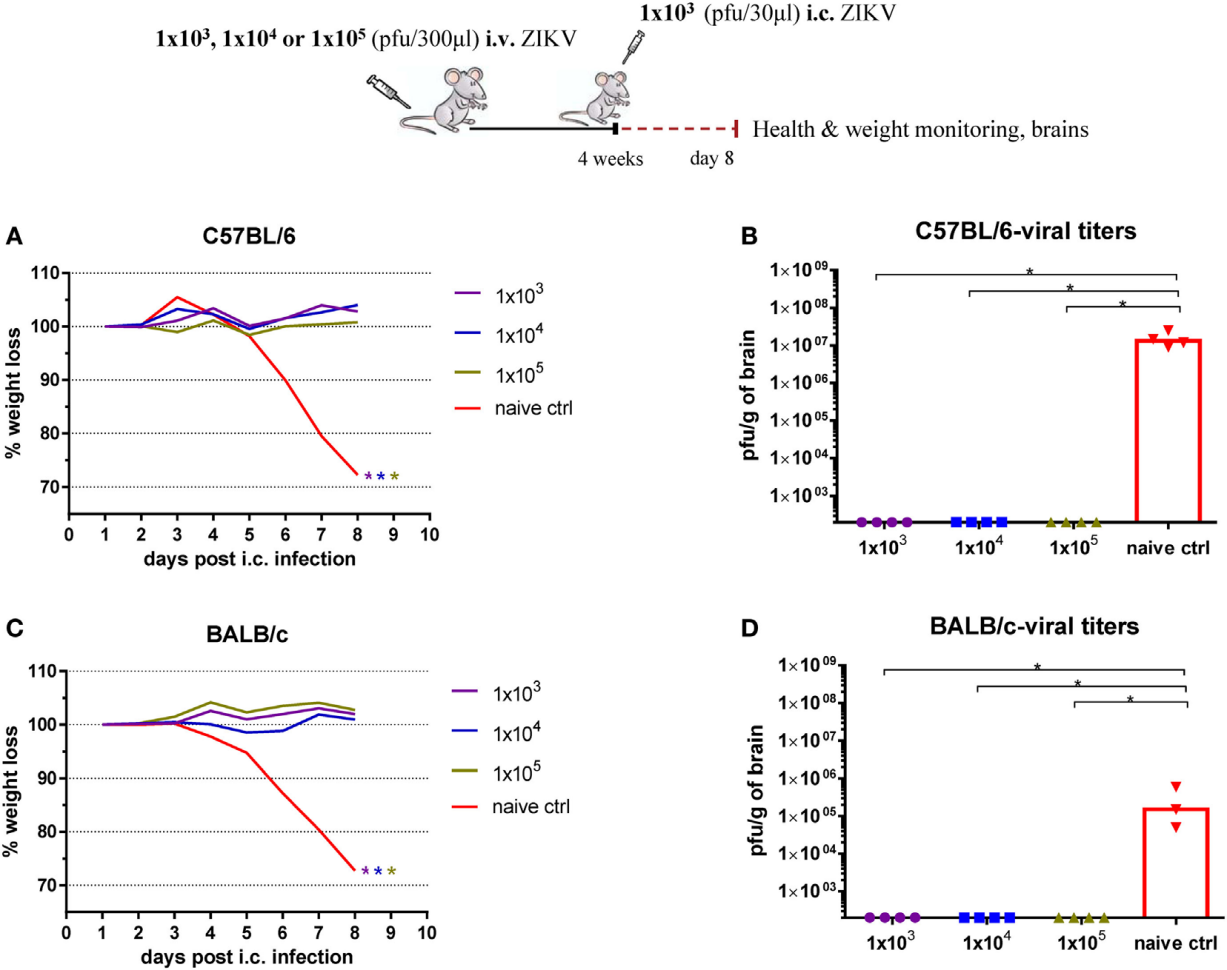
Determination of a suitable dose for i.v. immunization. Wild-type C57BL/6 **(A,B)** and BALB/c **(C,D)** mice were inoculated with 1 × 10^3^, 1 × 10^4^, or 1 × 10^5^ pfu Zika virus (ZIKV) i.v., and 4 weeks later, these mice and naïve controls were challenged with 1 × 10^3^ pfu ZIKV i.c. Mice were weighed daily **(A,C)**, and on day 7 post i.c. challenge, brains were removed and viral titers were measured by a plaque assay **(B,D)**. The detection limit for virus in the brain was 250 pfu/g organ. Each dot represents an individual animal and bars the medians of the groups. The weight curves **(A,C)** depict the group medians. *n* = 3–4 mice/group, *p < 0.05. For the weight curves, the color of the star denotes the infected group, which forms the basis for statistical comparison with naïve mice.

To investigate if such an asymptomatic “infection” might impact the outcome of an otherwise lethal i.c. infection with ZIKV, we infected WT C57BL/6 and BALB/c mice i.v. with three different doses of ZIKV (1 × 10^3^, 1 × 10^4^, or 1 × 10^5^ pfu) and 4 weeks later all mice received a lethal dose of ZIKV i.c. On day 7 post i.c., brains were removed and organ virus titers were determined by a plaque assay. We observed that all mice that had previously received the virus peripherally were able to preserve their body weight and prevent lethal disease by successfully controlling viral replication in their brains. By contrast, matched naive mice were unable to control the infection and experienced a severe weight loss associated with high viral loads in their brains on day 7 post i.c. challenge (Figures 2A,D).

**Figure 3:**
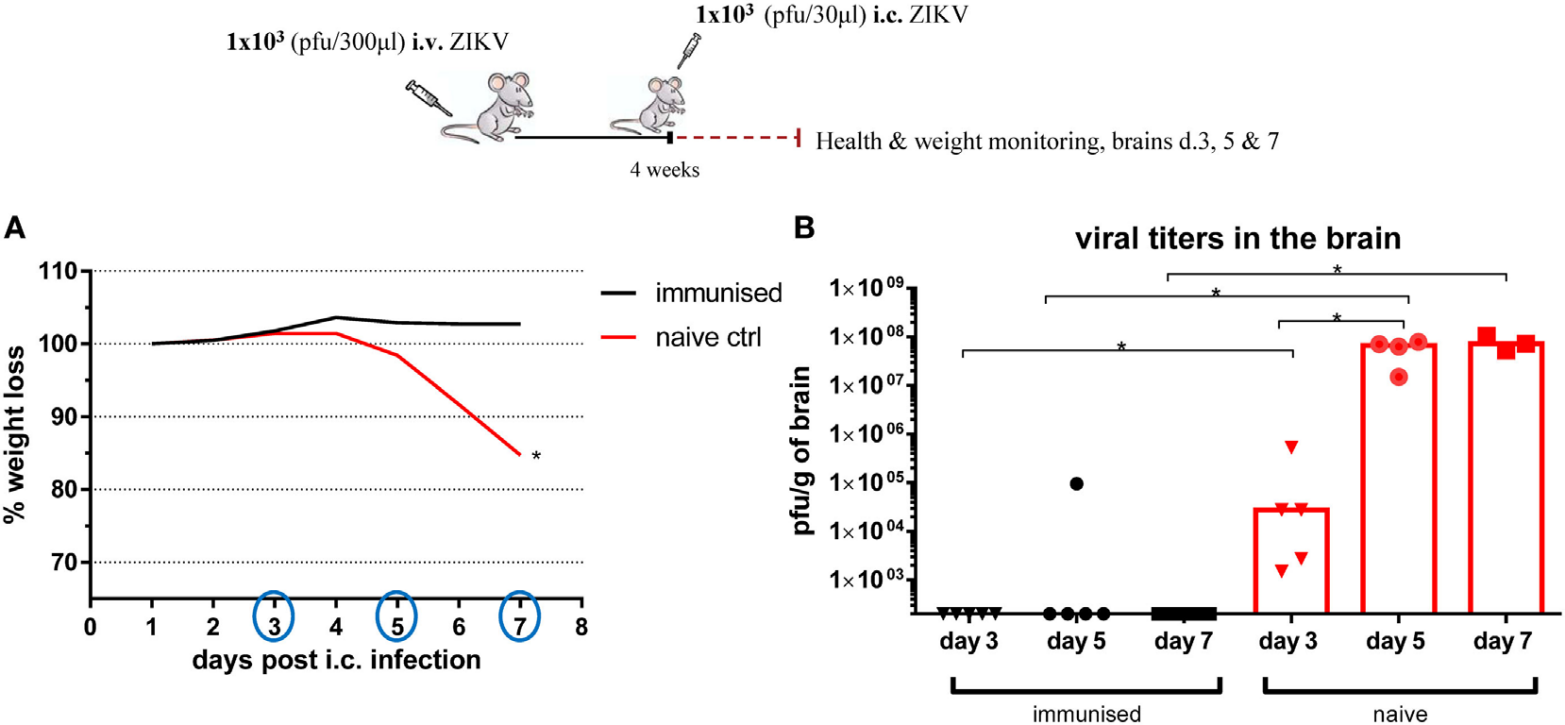
Kinetics of virus control after i.c. challenge. Wild-type C57BL/6 mice were inoculated with 1 × 10^3^ pfu Zika virus (ZIKV) i.v., and 4 weeks later, these mice and naïve controls were challenged with 1 × 10^3^ pfu ZIKV i.c. Mice were weighed and monitored daily **(A)**, and on days 3, 5, and 7 post i.c. challenge, brains were removed and viral titers were measured by a plaque assay **(B)**. The detection limit for virus in the brain was 250 pfu/g organ. Each dot represents an individual animal and bars the medians of the groups. The weight curves depict the group medians. *n* = 3–5 mice/group, *p < 0.05.

The fact that even a relatively low (1 × 10^3^) dose of ZIKV given i.v. was able to induce full protection against an otherwise lethal i.c. challenge was interesting, and we wanted to further gage the efficiency of this protection. Thus, WT C57BL/6 mice were infected with 1 × 10^3^ ZIKV i.v., and 4 weeks later, these mice, along with a group of naive control mice, received a lethal i.c. challenge of ZIKV. Health and weight were monitored daily and brains were removed on days 3, 5, and 7 post i.c. challenge, and the levels of infectious virus in the CNS at these times were measured by a plaque assay (Figure 3). Unlike the situation in other organs, ZIKV replicated to high titers in the CNS of i.c. infected, naïve WT mice. Thus significant levels of virus were detected in the CNS already by day 3 post i.c. challenge, and even higher levels were measured by days 5 and 7 post i.c. challenge, coinciding with increased severity of the clinical disease. By contrast, mice peripherally inoculated with virus 4 weeks prior to i.c. challenge were not only able to clear the infection before onset of clinical symptoms, but already by day 3 after i.c. challenge, we could not find infectious virus in their brains.

**Figure 4:**
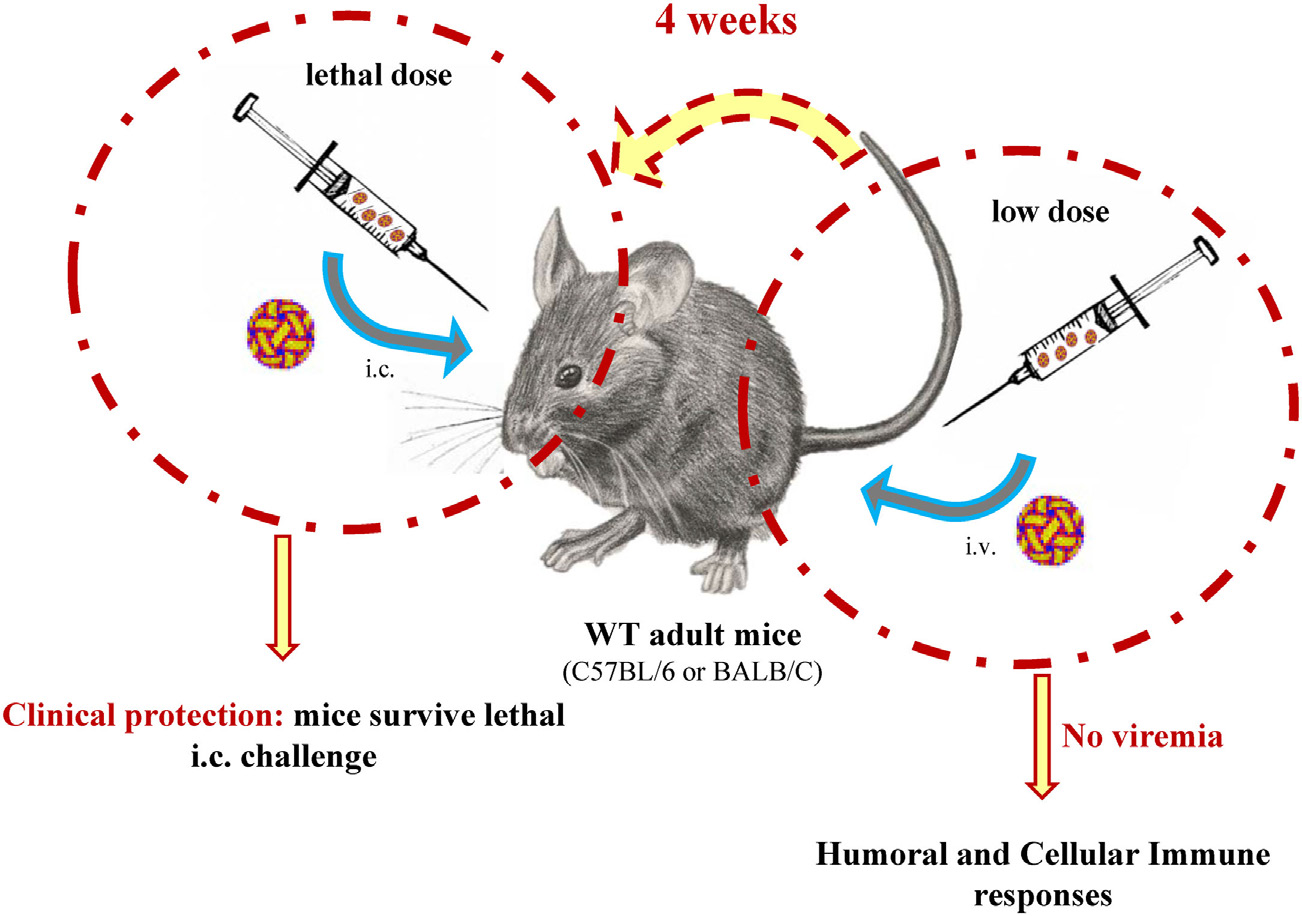
Graphical representation of our *in vivo* mouse model for Zika virus (ZIKV) infection. Mice are infected peripherally with low doses of ZIKV, and 4 weeks later, they are challenged i.c. with lethal doses of ZIKV.

Hence, we had established a model where strong protection against lethal i.c. infection is induced in WT mice through an asymptomatic primary i.v. infection with low doses of ZIKV (Figure 4). Remarkably, similar clinical protection was observed when the peripheral inoculation was performed *via* the subcutaneous route (Figure S1 in Supplementary Material).

### Primary Asymptomatic ZIKV Infection Induces Both Abs and CD8 T Cell Responses in WT Mice

The fact that peripheral administration of a low ZIKV inoculum leads to asymptomatic infection closely resembles the course of ZIKV infection in humans, which is mostly subclinical with only 20% of the infected people developing flu-like symptoms (30). Additionally, studies of human immune responses to ZIKV infection have revealed the induction of both B and T cell responses in the acute phase of infection (31). Therefore, we sought to investigate whether low doses of ZIKV could invoke humoral and/or cellular immune responses despite the lack of detectable infection in the peripherally inoculated WT mice.

**Figure 5:**
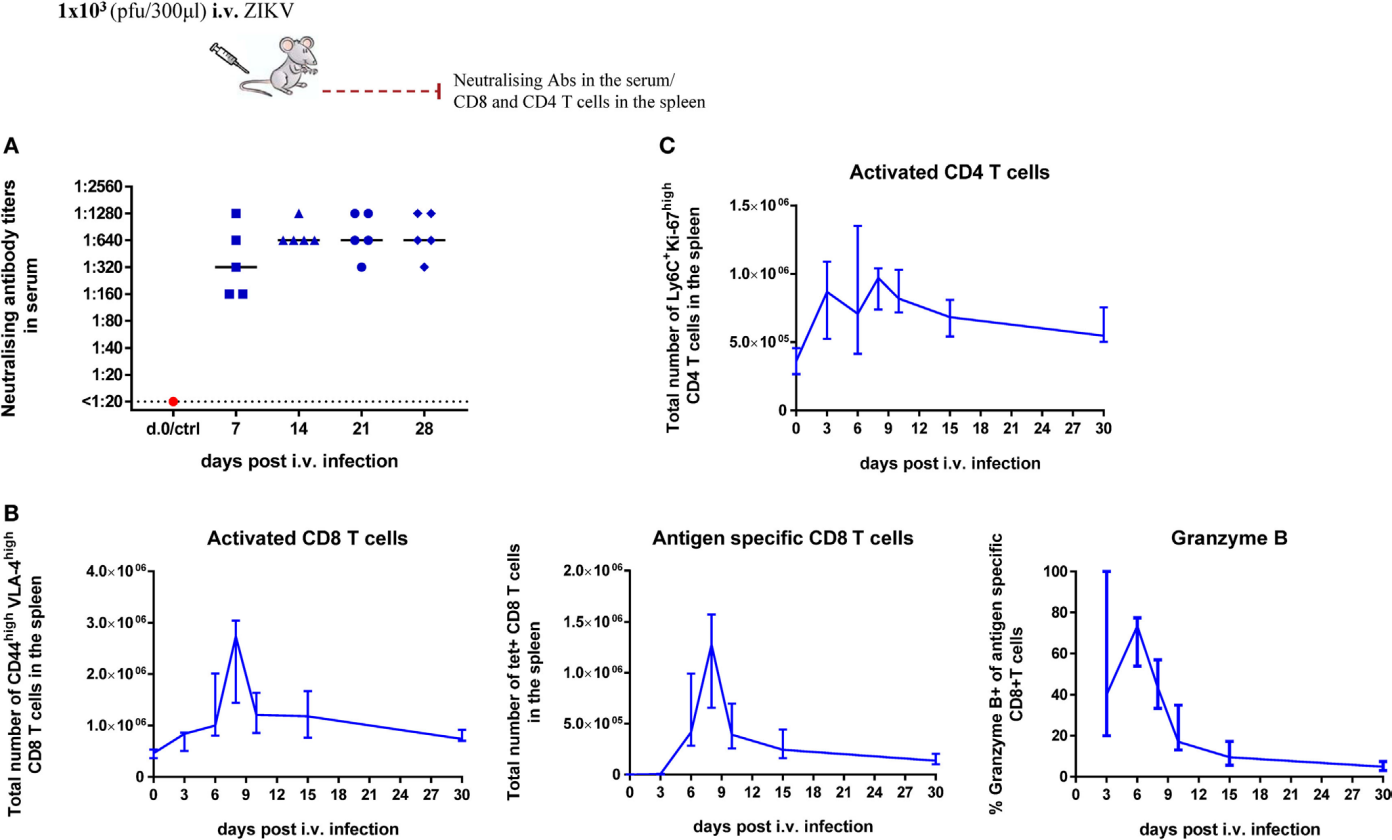
Cellular and humoral responses to Zika virus (ZIKV) infection in wild-type (WT) mice. WT C57BL/6 mice were inoculated with 1 × 10^3^ pfu ZIKV i.v. On days 7, 14, 21, and 28 post i.v. inoculation, sera were collected and ZIKV-specific Abs were determined by an *in vitro* plaque reduction assay. Each dot represents one mouse; bars denote group medians **(A)**. On days 3, 6, 8, 10, 15, and 30 post i.v. inoculation, spleens were harvested and total numbers of activated (CD44^high^VLA-4^high^) CD8 T cells, total numbers of antigen-specific [tetramer^+^ (H-2D^b^/E_294–302_)] CD8 T cells as well as the percentage of antigen-specific CD8 T cells producing granzyme B were determined by flow cytometry **(B)**. On same days as in panel **(B)**, splenocytes were stained with anti-CD4 and Th1 polarized (Ly-6C^high^) cycling (Ki-67^+^) CD4 T cells were enumerated. Panels **(B, C)** depict medians and ranges; *n* = 5–10 mice/time point **(C)**.

To that end, WT C57BL/6 mice were infected with 1 × 10^3^ pfu ZIKV i.v., and we assessed the levels of neutralizing Abs in the serum of infected WT mice on days 7, 14, 21, and 28 post i.v. infection by an *in vitro* plaque reduction assay. Briefly, serial dilutions of the serum were incubated with ZIKV and the reduction in plaque formation on Vero cells monolayers was measured and translated into neutralizing potency. We observed that the neutralizing potential of the Abs in the serum was already highly significant on day 7 post i.v.; it increased further on day 14 and remained at the same high level until at least day 28, the last day tested (Figure 5A).

Additionally, we assessed the ZIKV-induced T cell response in the spleen on days 3, 6, 8, 10, 15, and 30 post i.v. A CD8 T-cell response was detected by flow cytometric analysis using surface staining reflecting T-cell activation (VLA-4 and CD44) and/or tetramer staining for ZIKV-specific CD8 T cells alone or in combination with intracellular staining for granzyme B production (Figure 5B; for representative plots, see Figure S2 in Supplementary Material). We found that, shortly after ZIKV infection, numbers of activated CD8 T cells increased markedly and antigen-specific CD8 T cells directed toward a single viral epitope could account for about 50% of these cells. The response peaked between 6 and 8 days post i.v. and had decreased substantially by day 10 post i.v. As further evidence pointing to the effector function of these cells, we observed the presence of granzyme B in many of the cells, suggesting that they represented a population of activated CD8 T cells with the capacity to kill virus-infected cells (32). We also detected a significant CD4 T cell response; in this case we had no epitope-specific population to look for, so to increase the resolution regarding recently generated Th1 effector cells, we used a combination of the Th1 activation marker Ly-6C (33, 34) and expression of Ki-67. Again, we found a significant response peaking around 6–10 days after infection. Thus, despite very limited viral infection, a potent immune response was induced in low dose, i.v. infected mice.

### Fast and Long-Lasting Clinical Protection in WT Mice Experiencing a Primary Asymptomatic ZIKV Infection

Next, we wondered how early are mice protected by a primary asymptomatic ZIKV infection and how long does the protection last?

**Figure 6:**
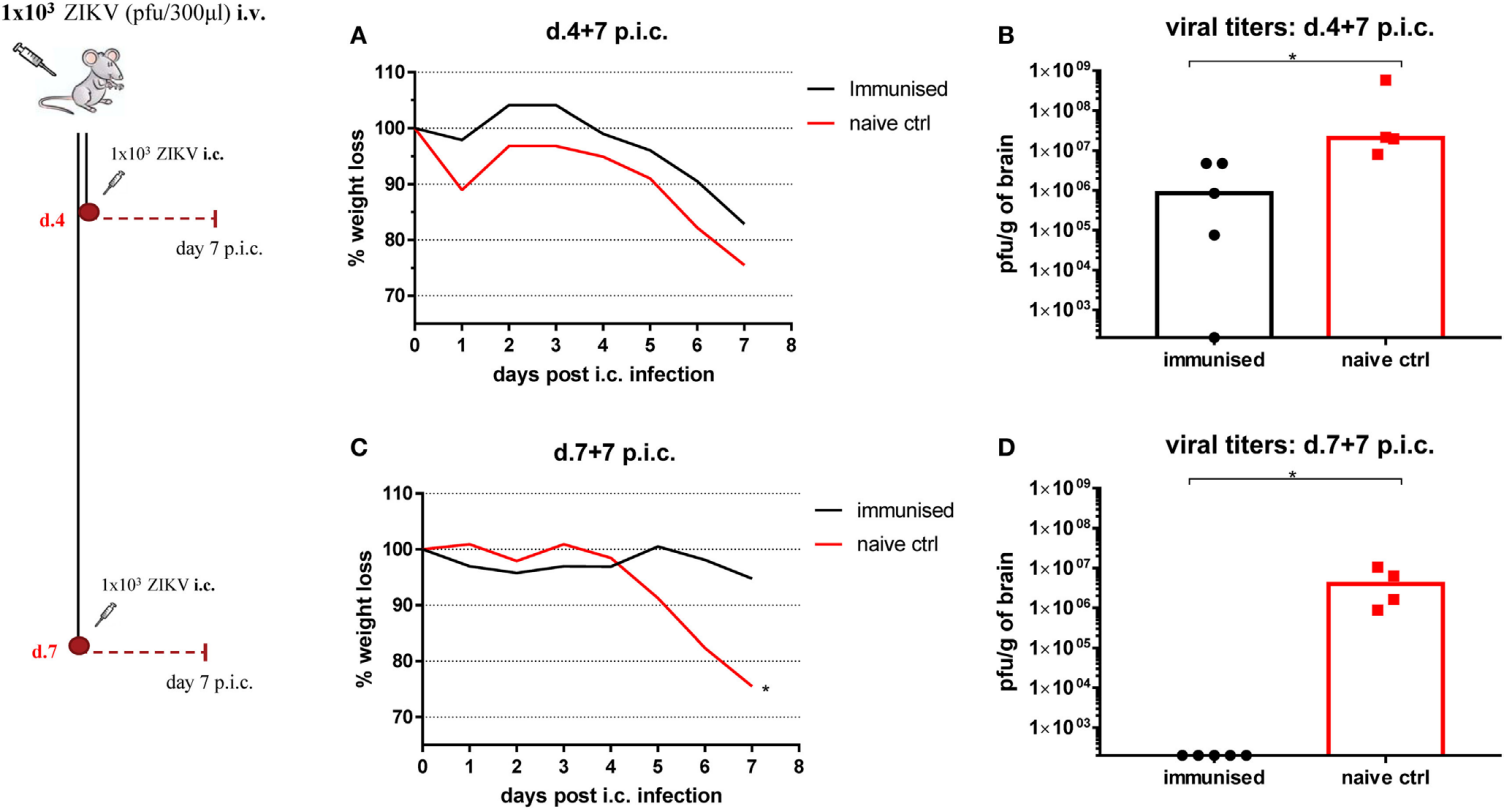
How quickly is protection induced? Wild-type C57BL/6 mice were inoculated with 1 × 10^3^ pfu Zika virus (ZIKV) i.v. and on days 4 **(A,B)** or 7 **(C,D)** post i.v. inoculation mice were challenged with 1 × 10^3^ pfu ZIKV i.c. Mice were weighed and monitored daily **(A,C)**, and on day 7 post i.c. challenge, brains were removed and viral titers were measured by a plaque assay **(B,D)**. Groups of naïve mice were used as negative controls. The detection limit for virus in the brain was 250 pfu/g organ. Each dot represents an individual animal, and bars represent the medians of the groups. The weight curves depict the group medians. *n* = 4–5 mice/group, *p < 0.05. The complete resistance observed 7 days post immunization was confirmed in a second experiment.

To investigate when protective immunity is obtained, WT C57BL/6 mice were immunized with 1 × 10^3^ pfu ZIKV i.v. and either 4 or 7 days later, they received a lethal challenge dose of ZIKV i.c. (Figure 6). We observed that the viral burden was significantly reduced in mice immunized 4 days prior to i.c. challenge while ZIKV was undetectable in the CNS of mice immunized 7 days prior to the challenge. As always, naive mice were incapable of controlling viral replication in the brain. This indicated the capacity of an asymptomatic ZIKV infection to induce a fully protective immune response within 1 week following immunization, roughly coinciding with the appearance of neutralizing Abs and a potent virus-specific CD8 T cell response (cf. Figure 5).

**Figure 7:**
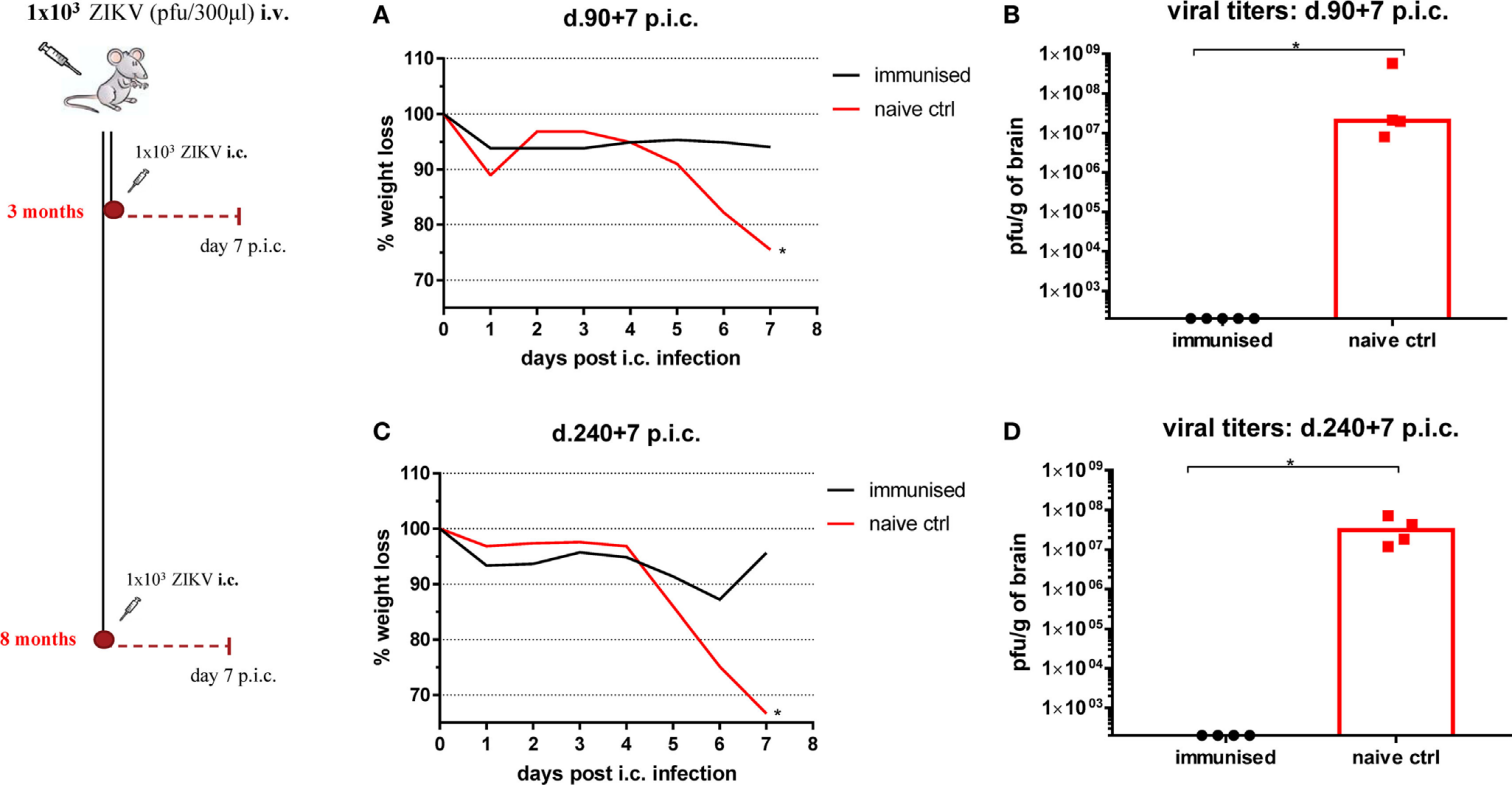
Longevity of protection. Wild-type C57BL/6 mice were inoculated with 1 × 10^3^ pfu Zika virus (ZIKV) i.v. and 3 **(A,B)** or 8 **(C,D)** months post i.v. inoculation mice were challenged with 1 × 10^3^ pfu ZIKV i.c. Mice were weighed and monitored daily **(A,C)**, and on day 7 post i.c. challenge, brains were removed and viral titers were measured by a plaque assay **(B,D)**. Mice were weighed and monitored daily, and on day 7 post i.c. challenge, brains were removed and viral titers were measured by a plaque assay. Groups of naïve mice were used as negative controls. The detection limit for virus in brain was 250 pfu/g organ. Each dot represents an individual animal, and bars represent the medians of the groups. The weight curves depict the group medians. *n* = 4–5 mice/group, *p < 0.05. Similar results were obtained in BALB/c mice (see Figure S2 in Supplementary Material).

To test how long after a single peripheral infection mice would still be protected against lethal infection, WT C57BL/6 mice were immunized as previously described and left for either 3 or 8 months prior to i.c. challenge (Figure 7). We saw that peripherally infected mice stayed clinically protected for at least 8 months and successfully controlled the lethal challenge at this point. In the same way, long-term protection (3 and 8 months post ZIKV immunization) was assessed also in WT BALB/c mice with similar results (Figure S3 in Supplementary Material).

To further gage the induced protection in our model, we tested how mice that were peripherally inoculated 4 weeks earlier would respond to i.c. challenge with much higher doses (1 × 10^5^ or 1 × 10^6^ pfu) of ZIKV. We observed that, for both C57BL/6 and BALB/c mice and for both challenge doses, no infectious virus could be isolated from the CNS 3 days after challenge, while substantial amounts of infectious virus was isolated from the CNS of matched naive controls (Figure S4 in Supplementary Material).

**Figure 8:**
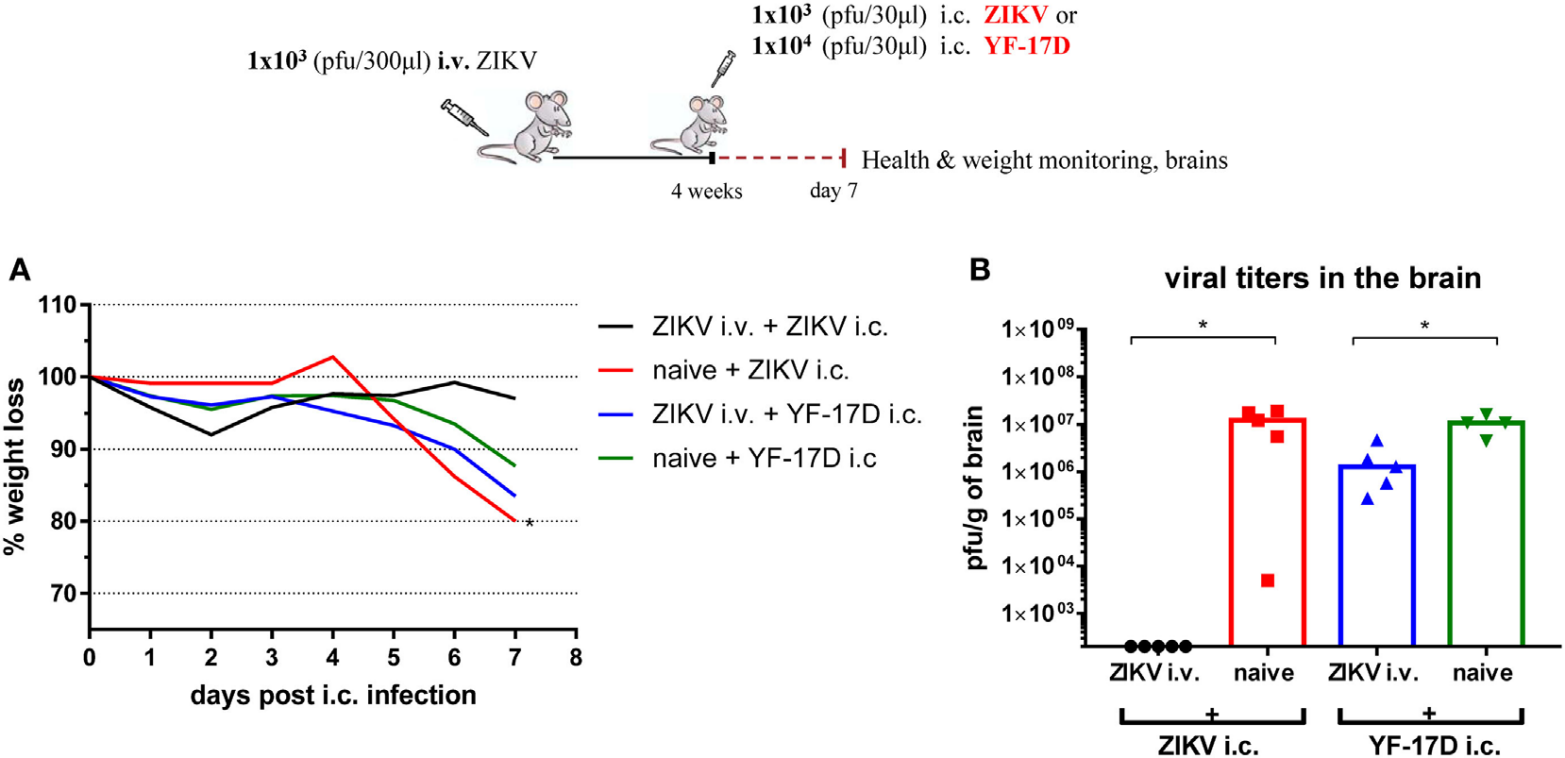
The induced protection is virus-specific. Wild-type C57BL/6 mice were inoculated with 1 × 10^3^ pfu Zika virus (ZIKV) i.v. and 4 weeks later were challenged i.c. with either 1 × 10^3^ pfu ZIKV or 1 × 10^4^ pfu YF-17D. Mice were weighed and monitored daily **(A)**, and on day 7 post i.c. challenge, brains were removed and viral titers were measured by a plaque assay **(B)**. Groups of naïve mice were used as negative controls. The detection limit for virus in the brain was 250 pfu/g organ for both viruses. Each dot represents an individual animal, and bars represent the medians of the groups. The weight curves depict the group medians. *n* = 4–5 mice/group, *p < 0.05, mice challenged with the same virus are compared. The results of one of two identical experiments are depicted.

Taken together, these results indicate that the immunity induced by an asymptomatic primary ZIKV infection comes fast is long lasting and is not breached even when high doses of ZIKV are administered i.c. Moreover, the induced protection is virus specific since i.c. challenge of ZIKV-immunized WT mice with lethal doses of YFV, a closely related flavivirus, was not efficiently controlled (Figure 8). In agreement with studies suggesting immunological cross-reactivity between different flaviviruses, we noticed that exposure to primary ZIKV infection prior to YFV i.c. challenge leads to a significantly reduced YFV load in the CNS—albeit not to the level required for full protection.

### Transfer of Immune Serum, but Not Immune Splenocytes, Protect Naive Mice From Lethal ZIKV i.c. Infection

Having established the ability of an asymptomatic primary ZIKV infection to induce effective and stable protection against i.c. challenge, we next wanted to shed light on which arm of the immune response was crucial in mediating this protection.

**Figure 9:**
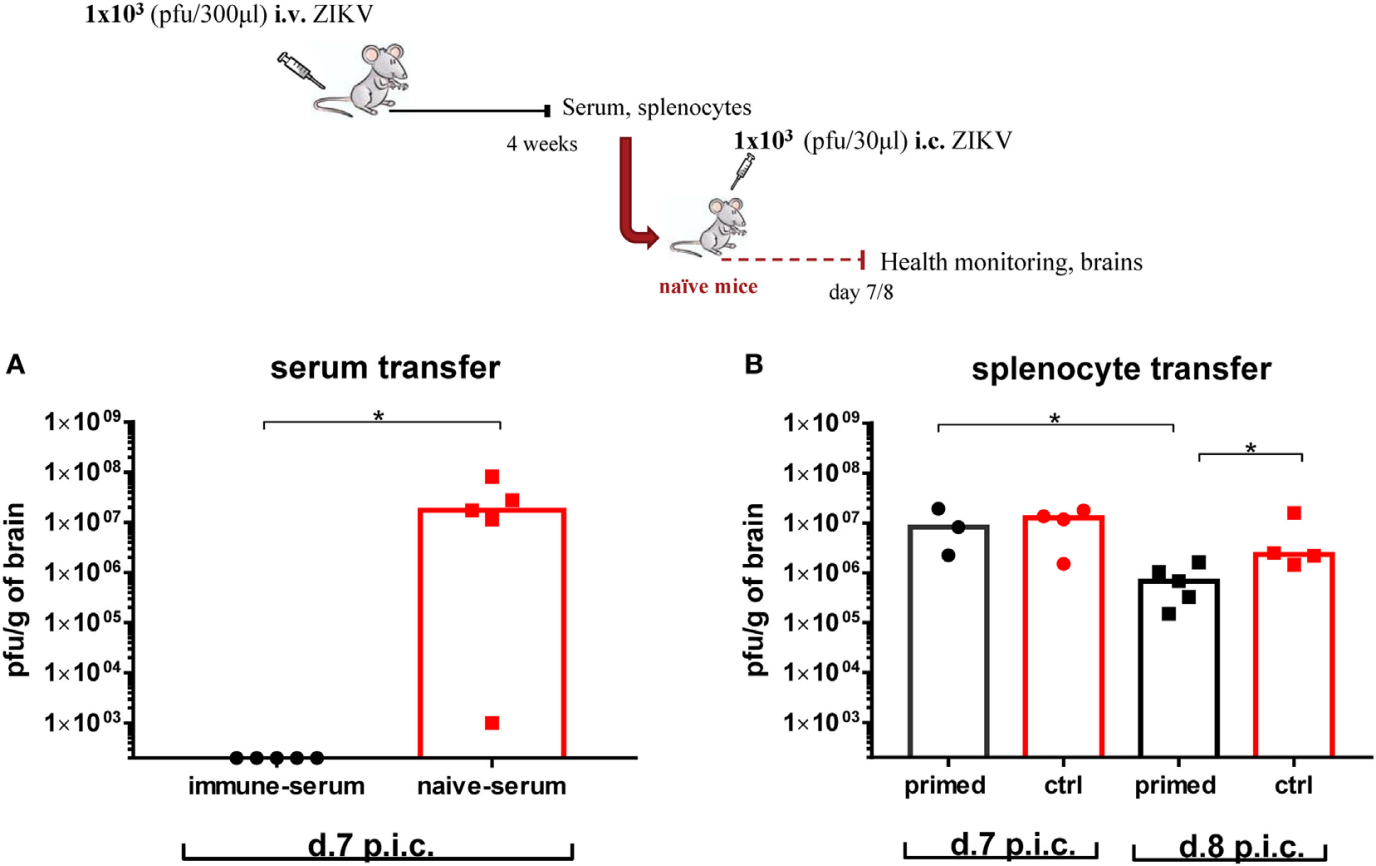
Immune serum transfer confers full protection against Zika virus (ZIKV), while primed splenocytes are marginally effective. Wild-type C57BL/6 mice were inoculated with 1 × 10^3^ pfu ZIKV i.v., and 4 weeks later, serum and splenocytes were harvested for adoptive transfer to naïve recipients. **(A)** Naïve mice received the immune serum 3 and 1 days prior to i.c. challenge with 1 × 10^3^ pfu ZIKV. On day 7 post i.c. challenge, brains were removed and viral titers were measured by a plaque assay. **(B)** Naïve mice received primed splenocytes 2 days prior to i.c. challenge with 1 × 10^3^ pfu ZIKV. Mice were weighed and monitored daily and on days 7 and 8 post i.c. challenge, brains were removed and viral titers were measured by a plaque assay. Groups of naïve mice, receiving serum or splenocytes (PBS) from naïve donors were included as negative controls. The detection limit for virus in the brain was 250 pfu/g organ. Each dot represents an individual animal, and bars represent the medians of the groups. *n* = 3–5 mice/group,*p < 0.05.

To evaluate the role of Abs in protection, WT C57BL/6 mice were immunized with 1 × 10^3^ pfu ZIKV i.v., and 4 weeks later, whole serum was harvested and transfused into naive recipient mice as described in Section “Materials and Methods.” Serum collected from naive mice was transferred to a control group. All recipient mice were challenged i.c. with a lethal dose of ZIKV 3 days after the first serum transfer (for details, see Materials and Methods), and their brains were removed on day 7 post i.c. (Figure 9A). While the mice receiving immune serum were fully protected against the i.c. challenge, the control mice receiving the naïve serum were not protected and developed high viral titers in the CNS on day 7 after challenge.

To address whether cellular components played a role in protection, we performed adoptive transfer of 7 × 10^7^ ZIKV-primed splenocytes (coming from the same immunized mice as above) into naive recipients 2 days prior to i.c. challenge; splenocytes collected from naive mice (or PBS) were transferred to a group of naive recipient mice as a control (Figure 9B). By contrast to primed-serum, transferred primed splenocytes only slightly reduced the viral load in the CNS and only when the effect was measured on day 8 post i.c. challenge. When the viral loads were measured on day 7 post i.c., no reduction in viral load was observed.

Overall, these results suggest that humoral immunity is sufficient for protection; however, a potential contribution of cellular immunity, particularly when specific Abs are lacking, cannot be excluded.

### Both B and T Cells Are Pivotal for the Protection Against a Lethal Outcome of i.c. Infection With ZIKV

**Figure 10:**
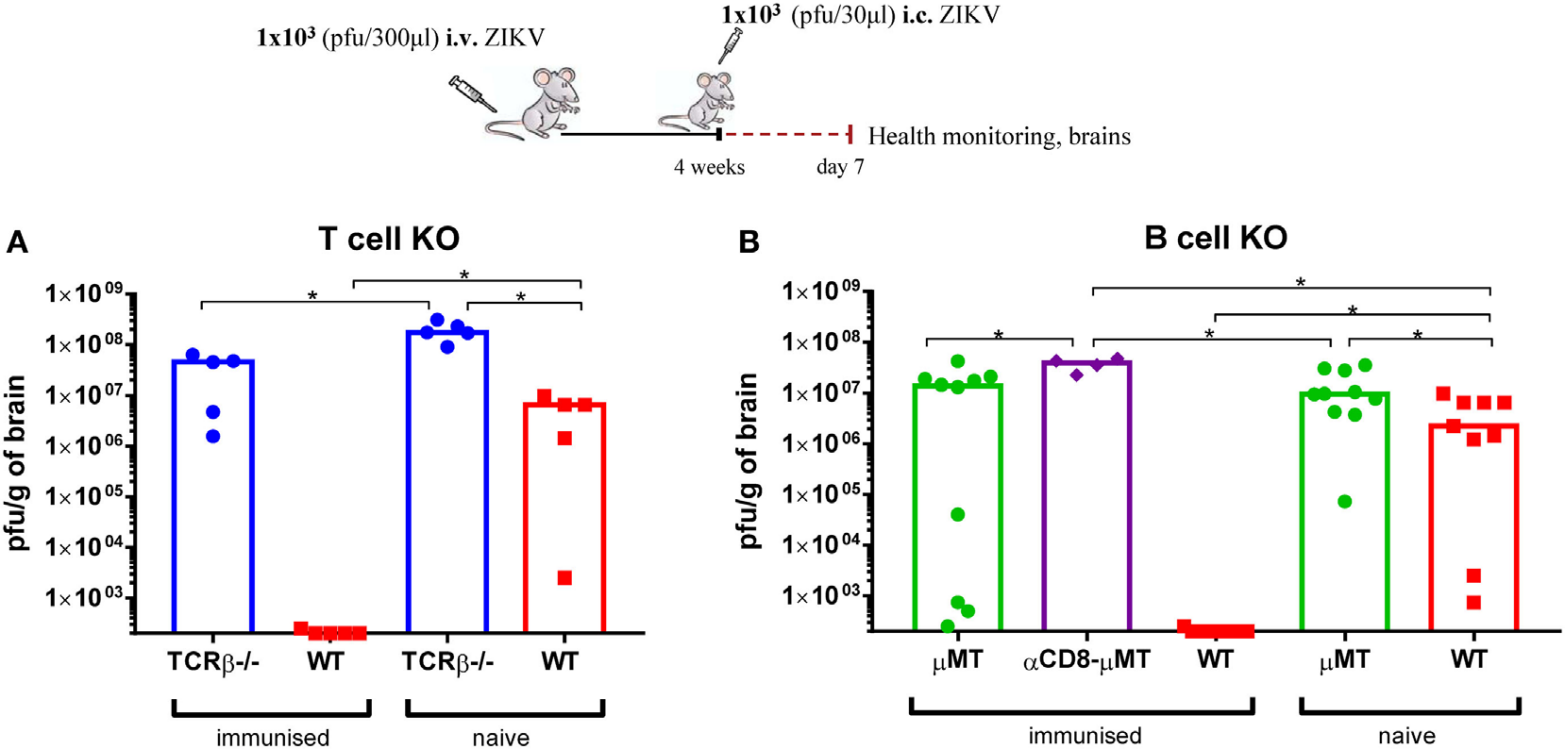
Both B and T cells are essential for induced protection against Zika virus (ZIKV). TCRβ-deficient (TCRβ^−/−^) **(A)** and B cell-deficient (μMT) **(B)** mice were inoculated with 1 × 10^3^ pfu ZIKV i.v. and 4 weeks later were challenged with 1 × 10^3^ pfu ZIKV i.c. A group of μMT-deficient mice were depleted of CD8 T cells (αCD8) prior to i.c. challenge. On day 7 post i.c. challenge, brains were removed and viral titers were measured by a plaque assay. For all deficient mice, a group of uninfected deficient mice was included for control. Additionally, wild-type (WT) C57BL/6 mice, inoculated with 1 × 10^3^ pfu ZIKV i.v., and naive uninfected mice were used as positive and negative controls, respectively. The detection limit for virus in the brain was 250 pfu/g organ. Each dot represents an individual animal, and bars represent the medians of the groups. *n* = 4–10 mice/group, *p < 0.05.

To further explore the role of adaptive immunity in protecting mice against a lethal outcome of i.c. challenge with ZIKV, B cell-deficient (μMT) and TCR αβ-deficient (TCRβ^−/−^) mice were compared to WT mice for their ability to resist a lethal ZIKV i.c. challenge when previously immunized with 1 × 10^3^ pfu ZIKV i.v. Naive mice of each mouse strain were included for control (Figure 10). We noted that, during the priming phase none of the immunodeficient mice displayed any obvious clinical signs of disease, which supports the notion that ZIKV control following peripheral inoculation can be mediated *via* innate immune mechanisms (35). Nevertheless, neither B nor T cell-deficient mice benefited from prior immunization and, unlike WT mice, were unable to clear ZIKV from the CNS when challenged i.c. This highlights the importance of B and T cells to mount protective immune responses that can save the mice from subsequent lethal challenge.

**Figure 11:**
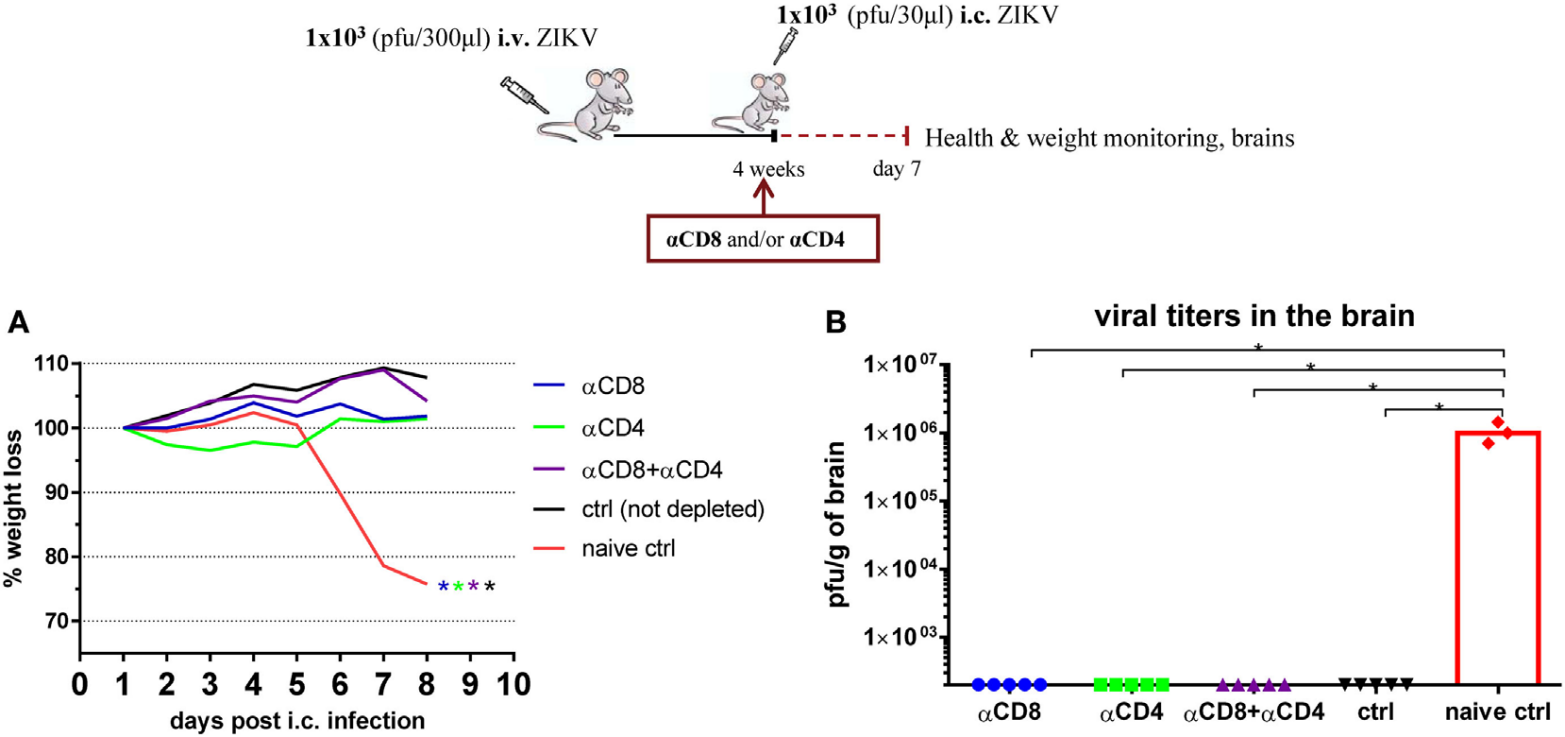
*In vivo* depletion of T cells. Wild-type C57BL/6 mice were inoculated with 1 × 10^3^ pfu Zika virus (ZIKV) i.v. and mice were challenged with 1 × 10^3^ pfu ZIKV i.c. On day −1, 1, and 4 post i.c., mice were depleted of CD8 (αCD8) and/or CD4 (αCD4) T cells. Mice were weighed and monitored daily **(A)**, and on day 7 post i.c. challenge, brains were removed and viral titers were measured by a plaque assay **(B)**. Groups of undepleted immunized and naïve mice were used as positive and negative controls, respectively. The detection limit for virus was determined as 250 pfu/g of brain. Each dot represents an individual animal and bars the medians of the groups. The weight curves depict the group medians. *n* = 3–5 mice/group, *p < 0.05. Immunized and naïve mice of the same strain is compared; the color of the star denotes the treated group used for statistical comparison. The results of one of two identical experiments are depicted.

Notably, when B cell-deficient mice were depleted of CD8 T cells immediately prior to i.c. challenge, the viral burden was found to be slightly, but significantly higher than the viral load in the brains of undepleted mice (Figure 10). By comparison, in WT mice where Ab production is intact, depletion of CD8 and/or CD4 T cells did not alter the outcome of i.c. challenge, and all depleted immunized mice seemed equally efficient at clearing ZIKV from the CNS (Figure 11). Therefore, given that B cell-deficient mice are unable to produce Abs, these results support the notion that CD8 T cells may contribute to virus control in the absence of specific Abs.

It is also worth pointing out that primary adaptive immune components appear to play a role in the course of infection following i.c. challenge in naive mice, since both naïve B and T cell-deficient mice displayed significantly higher viral loads in the CNS than matching WT mice.

### CD4 T Cells Are Required for Protection Against Subsequent i.c. Infection, While CD8 T Cells Might Function as a Back-up Mechanism

Even though *in vivo* depletion of T cells prior to i.c. challenge did not impair the level of protection in immunized WT mice, that finding does not exclude a significant contribution of T cells in the priming/immunization phase or as tissue resident effector cells. Therefore, we next sought to investigate the role of T cells using a range of genetically modified mice with targeted immune defects within the T cell compartment.

**Figure 12:**
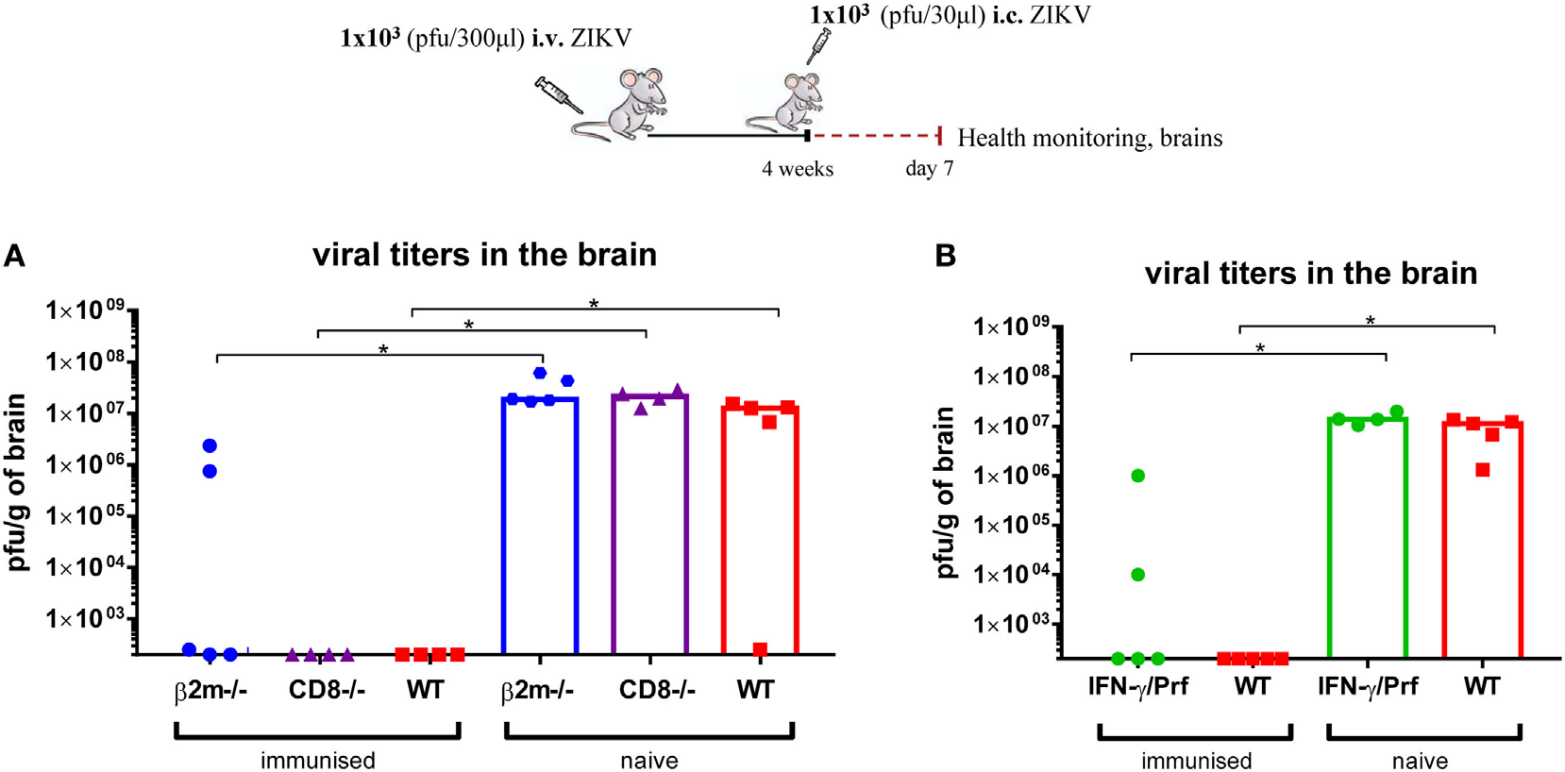
CD8 T cells are not essential for protection against Zika virus (ZIKV). β2-microglobulin-deficient (β2m ^−/−^) and CD8α-deficient (CD8^−/−^) **(A)** or IFN-γ/perforin double-deficient (IFN-γ/Prf^−/−^) **(B)** mice were inoculated with 1 × 10^3^ pfu ZIKV i.v. and 4 weeks later were challenged with 1 × 10^3^ pfu ZIKV i.c. On day 7 post i.c. challenge, brains were removed and viral titers were measured by a plaque assay. For all deficient mice, a group of uninfected deficient mice was included for control. Additionally, wild-type (WT) C57BL/6 mice, inoculated with 1 × 10^3^ pfu ZIKV i.v., and naive uninfected mice were used as positive and negative controls, respectively. The detection limit for virus in the brain was 250 pfu/g organ. Each dot represents an individual animal, and bars represent the medians of the groups. *n* = 4–5 mice/group, *p < 0.05.

We started by studying the contribution of CD8 T cells. We administered 1 × 10^3^ pfu ZIKV i.v. to CD8α-deficient, β2-microblobulin-deficient, and perforin/IFN-γ double-deficient mice, and 4 weeks later we compared the outcome of i.c. challenge to that in WT mice. Naive mice of each mouse strain were included for control. We observed that immunized CD8-deficient and the majority of β2-microglobulin-deficient mice were as efficient as WT mice in controlling a lethal ZIKV i.c. infection (Figure 12). The inability of the few immunized β2-microblobulin-deficient mice to completely clear the infection could be attributed to the fact that these mice often display a substantially reduced level of Abs (36). Thus, again the results were consistent with a role of T cells when Ab levels are not optimal. Interestingly, some immunized perforin/IFN-γ double-deficient mice also seemed to fail to completely clear the i.c. infection by day 7 post i.c. challenge, suggesting that effector cells (T cells and/or NK cells) might also play a minor role in controlling the infection of B-cell replete mice (Figure 12).

**Figure 13:**
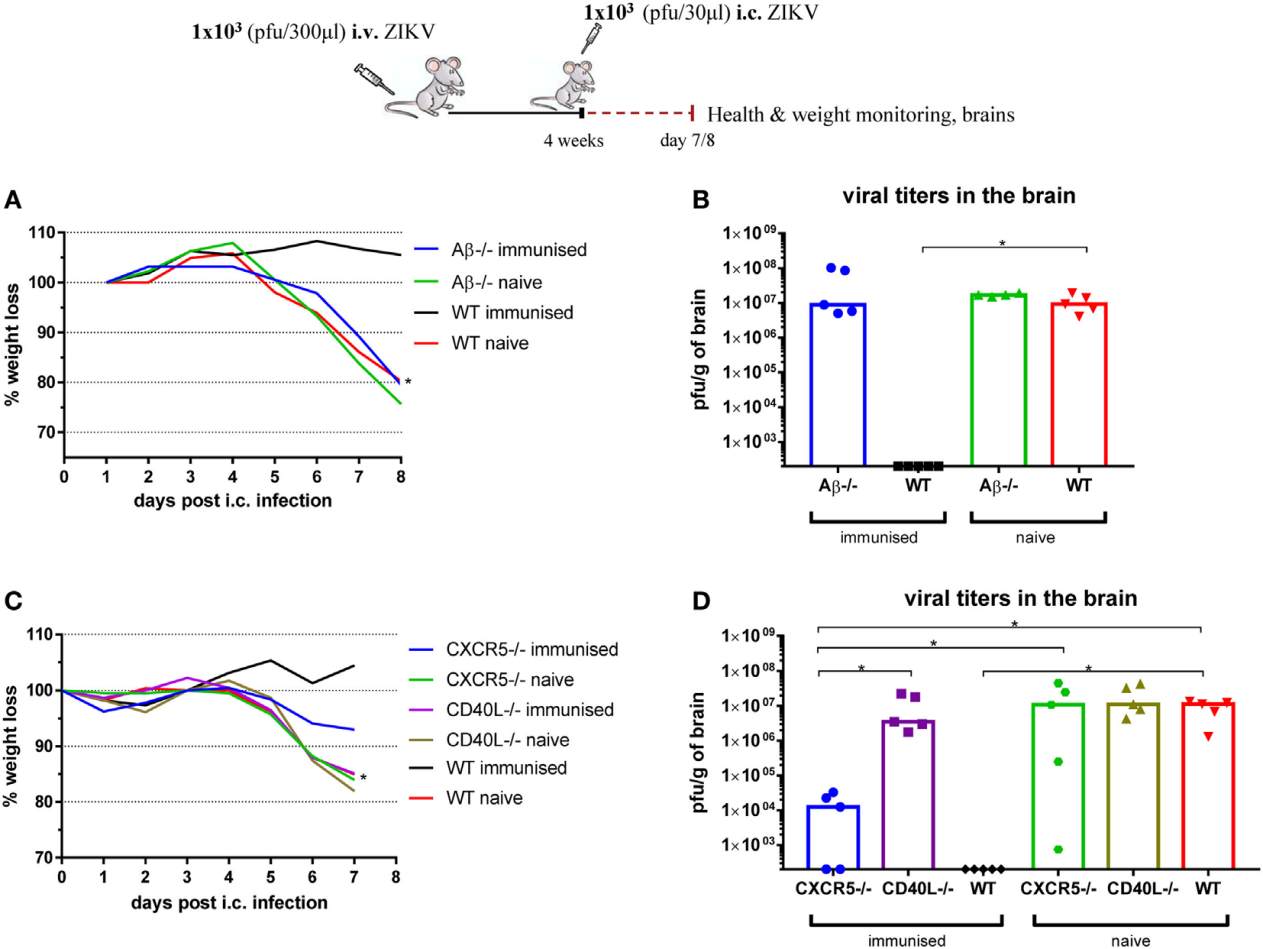
CD4 T cells are essential for protection against Zika virus (ZIKV). MHC class II-deficient (Aβ^−/−^) **(A,B)** or CXCR5-deficient (CXCR5^−/−^) and CD40L-deficient (CD40L^−/−^) **(C,D)** mice were inoculated with 1 × 10^3^ pfu ZIKV i.v. and 4 weeks later were challenged with 1 × 10^3^ pfu ZIKV i.c. Mice were weighed and monitored daily **(A,C)**, and on day 7 post i.c. challenge, brains were removed and viral titers were measured by a plaque assay **(B,D)**. For all deficient mice, a group of uninfected deficient mice was included for control. Additionally, wild-type (WT) C57BL/6 mice, inoculated with 1 × 10^3^ pfu ZIKV i.v., and naive uninfected mice were used as positive and negative controls, respectively. The detection limit for virus in the brain was 250 pfu/g organ. Each dot represents an individual animal. The results illustrate the group medians. The weight curves depict the group medians. *n* = 4–5 mice/group, *p < 0.05. For the weight curves, immunized and naïve mice of the same strain have been compared, and the color of the star marks the immunized group used in the statistical comparison.

Regarding the contribution of CD4 T cells, we administered 1 × 10^3^ pfu ZIKV i.v. to MHC-II-deficient (Aβ^−/−^), CD40L-deficient, and CXCR5-deficient mice, and 4 weeks later, we compared the outcome of i.c. challenge to that in WT mice. Again, naive mice of each mouse strain were included for control (Figure 13). We observed that the absence of CD4 T cells (in Aβ^−/−^ mice) as well as their inability to provide T-cell help (CD40L^−/−^) can severely impair effective viral clearance following i.c. challenge, and both immunodeficient strains displayed levels of replicating virus in the CNS similar to the naive control groups. Immunized CXCR5-deficient mice experienced a milder infection following lethal i.c. challenge and did not display the same weight loss as the naive groups (data not shown); however, substantial viral replication was still found in the CNS of some of the former mice, which implies the need for efficient germinal center formation and isotype switching for optimal protection.

An overview of the knockout mouse strains used in this study and the level of protection induced by peripheral priming preceding lethal i.c. challenge are shown in Table S1 in Supplementary Material.

## Discussion

Even though ZIKV has been known for more than half a century, a comprehensive analysis of the immune mechanisms that protect most immunocompetent hosts from developing severe disease has not been carried out before. In mice, little ZIKV replication can be detected in most organs unless early type I IFN activity is impaired, and mice treated with IFNAR-blocking Abs or equivalent genetically deficient mice are currently the favored model for studying aspects of ZIKV infection in mice (20–22). However, since type I IFN impacts not only the innate but also the adaptive immune response (37, 38), type I IFN-deficient models are not optimally suited to provide an in-depth analysis of the natural protective adaptive immune response. Therefore, based on our previous experiences with mice infected with the closely related YFV (24), we predicted that direct i.c. challenge might represent a means to bypass this early innate defense and induce clear virus associated disease that could be studied. Indeed, this prediction was fulfilled in both mouse strains tested (BALB/c and B6). Remarkably, peripheral inoculation with a low dose of the virus into type I IFN replete mice, which did not lead to detectable production of infectious virus in the spleen, nonetheless, did induce a protective host response. This made it possible for us to use fully type I IFN replete mice to study, which components of the adaptive immune system are essential to prevent reinfection with ZIKV.

As also recently reported by others (25, 39, 40), we found that peripheral ZIKV infection in WT mice induces both a humoral and a T-cell-mediated immune response. More importantly, fully competent, peripherally infected mice challenged i.c. 30 days later showed little if any evidence of disease, while matching naïve mice consistently became severely ill and had to be euthanized for humane reasons around day 8 post infection. For subsequent interpretation of the results in gene-targeted mice, it should be pointed out that the induced disease did not appear to reflect immunopathology, since similar severe disease was observed in the naïve mice of all the tested mouse strains including TCRβ and CD8a knockouts. This is at variance to a recent report which claims an important role for CD8 T cells in causing hind-limb paralysis (41). Analysis of the virus level in the CNS of i.c. infected immune mice failed to reveal detectable virus replication in the brain of most of the immune mice at any time point after challenge, and even a 100- to 1,000-fold increase in the challenge dose did not lead to an early viral breakthrough, suggesting a very solid state of antiviral immunity in i.v. infected mice. A similar resistance to i.c. infection was observed in mice primed *via* the s.c. route, which supports the assumption that limited peripheral infection results in solid immunity. Protection against infection with the unrelated flavivirus, YFV was significant, but limited, consistent with the notion that there is some, but incomplete cross-reactivity between these two viruses. By gradually increasing the interval between peripheral immunization and subsequent i.c. challenge, we could demonstrate that protective immunity was induced between 4 and 7 days after virus inoculation, coinciding with the appearance of both neutralizing Abs and virus-specific CD8 T cells, and in both mouse strain tested clinical protection was very stable over time; thus, even 8 months after priming, mice challenged i.c. effectively controlled the infection and did not develop evidence of disease. Adoptive transfer of serum from immunized mice fully protected against clinical disease, while splenocytes from the same donors only marginally impacted virus levels in the CNS at day 7 after challenge of the recipients. This indicated that humoral immunity was most important in otherwise intact hosts, but cellular immunity might also play a role, perhaps as a backup when few specific Abs are present in the circulation at the time of infection.

For a more in-depth analysis of the adaptive immune response to Zika we used various gene-targeted mice, which were first primed through peripheral infection and challenged i.c. 30 days later. None of the immunodeficient mice developed obvious signs of disease during the priming phase indicating that none of the impaired gene functions individually were mandatory in order to survive a peripheral ZIKV challenge. However, neither TCR αβ T-cell-deficient nor B-cell-deficient mice were able to control subsequent infection of the CNS indicating that both cell types were required to resist i.c. infection. In B-cell-deficient mice, we could further show that CD8 T cells could serve as backup in the absence of circulating Abs, as indicated by a significantly higher level of virus in the CNS of CD8 T-cell depleted, B-cell-deficient mice. This is in contrast to the situation in WT mice where Abs are present prior to challenge. In this case, it was found that neither CD4 nor CD8 T cells are critically required in the effector phase, based on depletion of these cell subsets individually or combined immediately before i.c. challenge.

It should perhaps be noted that generally viral loads in the CNS of naïve mice day 7 post i.c. challenge tended to be slightly higher in the most severely immunocompromised, gene-targeted mice compared to naïve WT mice. This could reflect that components of the adaptive immune system start to play a role even in unimmunized WT mice around this time point. Consistent with this possibility, we observed significant T-cell and monocyte/macrophage infiltration in previously unimmunized WT mice at the time when we have to euthanize them based on the severity of their clinical symptoms (Nazerai et al., manuscript in preparation).

Eliminating the T cells prior to the challenge does not address their role during priming or even as tissue resident memory cells since these might not be efficiently depleted by the Ab injection. Therefore, to further map the role of T cells, we immunized mice with more specific deficiencies within their T-cell compartment. Overall, CD8 KO mice and most beta2-microglobulin-deficient mice controlled the challenge infection quite well; the few exceptions among the beta2-microglobulin-deficient mice could reflect the well-known fact that these mice not only lack CD8 T cells but also catabolize their Abs faster than normal mice and for that reason carry lower levels of Abs in addition to their T-cell defect (42). Therefore, CD8 T cells might play a role when Abs levels are suboptimal. The finding that a few perforin/IFN-γ double-deficient mice display a similar phenotype could also point to a back-up function of virus-specific effector T cells, as it is also indicated by our findings in CD8-depleted B-cell-deficient mice.

Consistent with a key role for B cells and antibodies, mice that lack CD4 T cells or the capacity to interact with B cells were found to be markedly impaired in their capacity to resist the i.c. challenge. Obviously, we cannot be certain that the requirement for CD4 T-cell help only pertains to the B cell response, but given the minor role of effector CD8 T cells, and the fact that effector CD4 T cells were not found to be required during the challenge, lack of help to the B cells is by far the most likely interpretation of the results. Also the observation that some CXCR5-deficient mice are impaired in their ability to control the challenge, despite the fact that germinal center formation and isotype switching may also occur to some extent in these mice (43), strongly support the idea that help to B cells and efficient germinal center formation is important for the development of optimal resistance to reinfection. Interestingly, this is in contrast to the situation in YFV immunized mice where CXCR5 deficiency did not impact resistance to i.c. challenge with the homologous virus (24).

In conclusion, using a peripheral prime, i.c. challenge approach, we have been able to establish a murine ZIKV challenge model in type I IFN replete mice. Others have also studied ZIKV infection in WT mice, but either the mechanism of protection, particularly the role of abs, was not explored (25, 39, 40) or newborn animals known to be deficient in their capacity for a type I IFN response were used (44, 45). Another important difference is that we have used low doses (10^3^ pfu) of virus i.v. or s.c. for immunization of the animals, not intraperitoneal infection with relatively high doses (≥10^4^ pfu). The current model is well suited for analysis of the natural adaptive immune response to ZIKV as well as for early, preclinical evaluation of potential vaccine candidates. In this study, we focused on the adaptive immune mechanisms underlying resistance to reinfection with ZIKV. Our results point to a critical role for humoral immunity, even though the details of how the Abs work *in vivo* is still unknown. This conclusion is in agreement with some of the first reports on vaccination against ZIKV (46, 47), but not with more recent reports, which have emphasized the role of CD8 T cells (21, 39, 40). According to our findings, CD8 T cells do make a minor contribution, but it is also very clearly documented that they are not pivotal in mice with sufficient preformed specific Abs. Consequently, an efficient prophylactic vaccine against ZIKV infection needs to induce at least a potent Ab response. Probably, a vaccine would work even more efficiently if it also induced a CD8 T cell response that could provide additional immunity in situations where Abs levels are insufficient for complete protection. Both our current results and recent results from other groups using type I IFN-deficient models (21, 39, 40) support this conclusion. It is also of considerable interest to note that asymptomatic, peripheral infection with ZIKV suffices to induce long-lasting protective immunity. Even though infection of the fetus has not been addressed in this study, the solid immunity observed following low dose peripheral infection strongly suggests that only the primary infection carries a real risk for fetal malformation. The implications of this prediction is that once a human population has thoroughly been exposed to ZIKV, most would eventually become resistant to subsequent infections, and the frequency of malformations would decline markedly. Even those uninfected might be protected through herd immunity unless the virus is able to establish and maintain itself in a suitable non-human host. Only in the latter case will a vaccine be required in the long run.

## Ethics Statement

Experiments were conducted in accordance with national Danish guidelines (Amendment # 1306 of November 23, 2007) regarding animal experiments as approved by the Danish Animal Experiments Inspectorate, Ministry of Justice, permission number 2015-15-0201-00623.

## Author Contributions

SB, AS, JC, and AT designed the study. LN, ASS, and PR performed the experiments and analyzed the data. LN, SB, AS, JC, and AT interpreted the data. LN and AT drafted the manuscript. All authors approved the final version.

## Conflict of Interest Statement

The authors declare that the research was conducted in the absence of any commercial or financial relationships that could be construed as a potential conflict of interest.
