# Supplementary material for: A New In Vivo Model to Study Protective Immunity to Zika Virus Infection in Mice With Intact Type I Interferon Signaling
Source: Front Immunol. 2018 Mar 22;9:593. doi: 10.3389/fimmu.2018.00593 (PMC5874300; doi:10.3389/fimmu.2018.00593)
Supplement: Supplementary file 1 [file presentation_1.PDF]

Electronic Supplementary Material

A new *in vivo* model to study protective immunity to Zika virus infection in mice with intact type I interferon signalling

Loulieta Nazerai, Amalie Skak Schøller, Peter Overbeck Sharma Rasmussen, Søren Buus, Anette Stryhn, Jan Pravsgaard Christensen and Allan Randrup Thomsen*

Department of Immunology and Microbiology, University of Copenhagen, Copenhagen, Denmark

*to whom correspondence has to be sent: Athomsen@sund.ku.dk

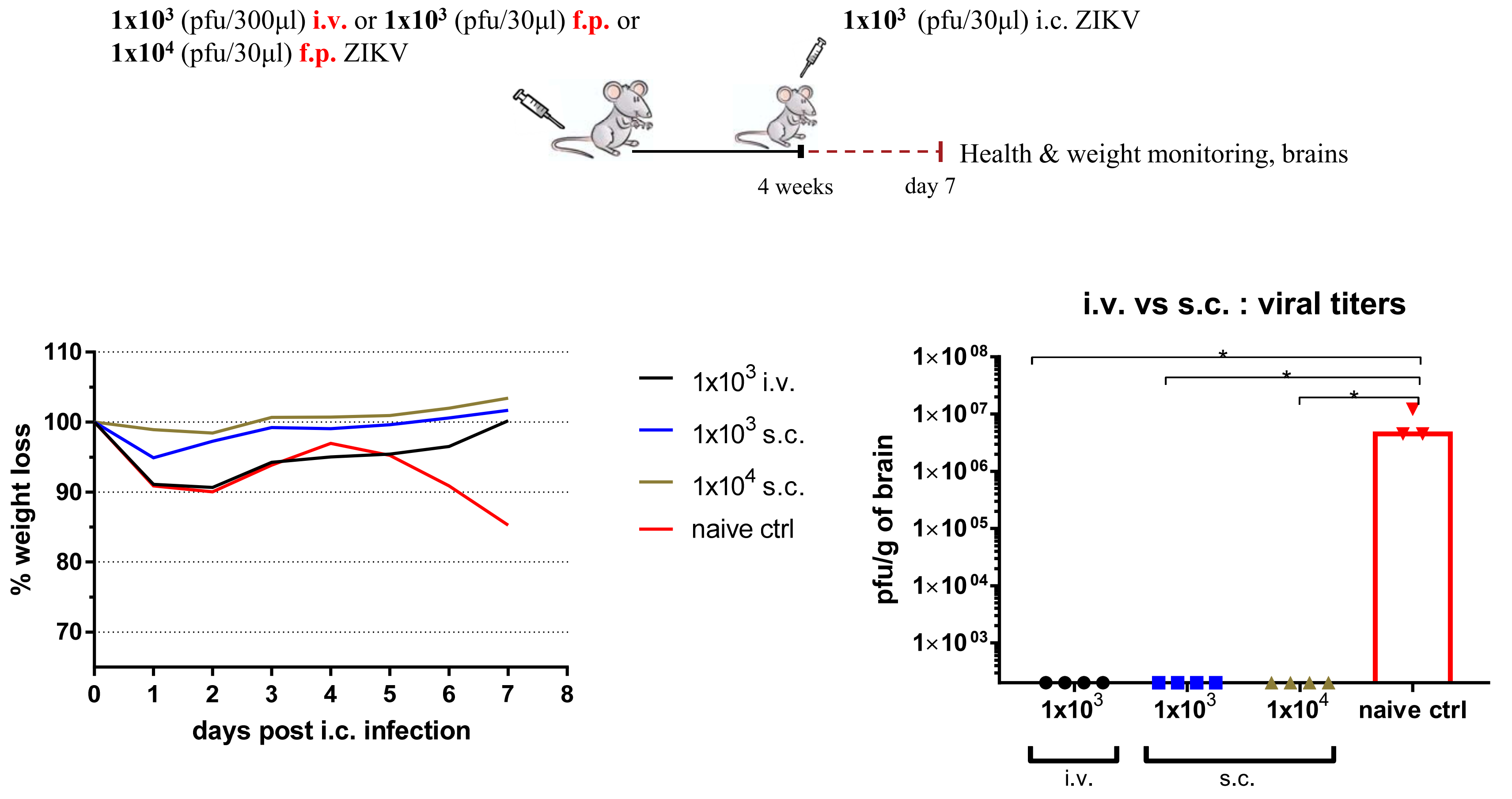

Supplementary figure 1: Subcutaneous ZIKV administration also confers clinical protection. WT C57BL/6 mice were inoculated i.v. with $1x10^3$ pfu ZIKV, subcutaneously (s.c.) with $1x10^3$ pfu ZIKV or s.c. with $1x10^4$ pfu ZIKV and 4 weeks later were challenged with $1x10^3$ pfu ZIKV i.c. Mice were weighed and monitored daily and on day 7 post i.c. challenge brains were removed and viral titers were measured by a plaque assay. A group of naïve mice was used as negative control. The detection limit for virus in the brain was 250 pfu/g organ. Each dot represents an individual animal and bars the medians of the groups. The weight curves depict the group medians. *$p < 0.05$.

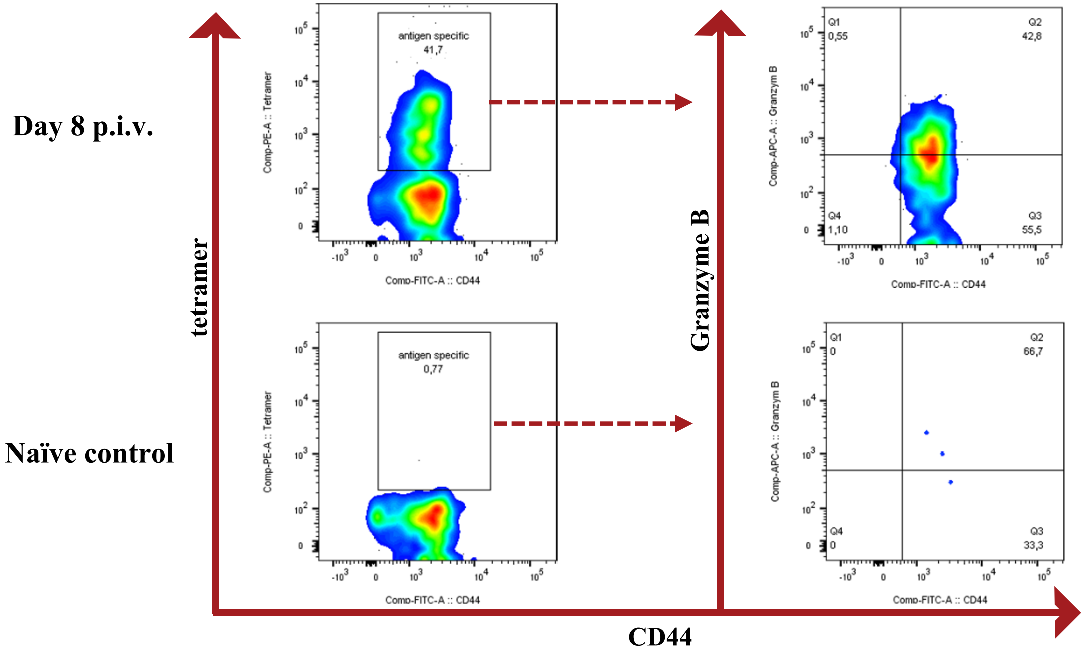

Supplementary figure 2: Representative flow cytometry plots for Zika activated CD8 T cells. Cells have been gated for CD8 T cells, and tetramer^{+}CD8 (tet^{+}CD8) T cells and tet^{+}CD8 T cells producing granzyme B on day 8 post i.v. infection with Zika virus are depicted.

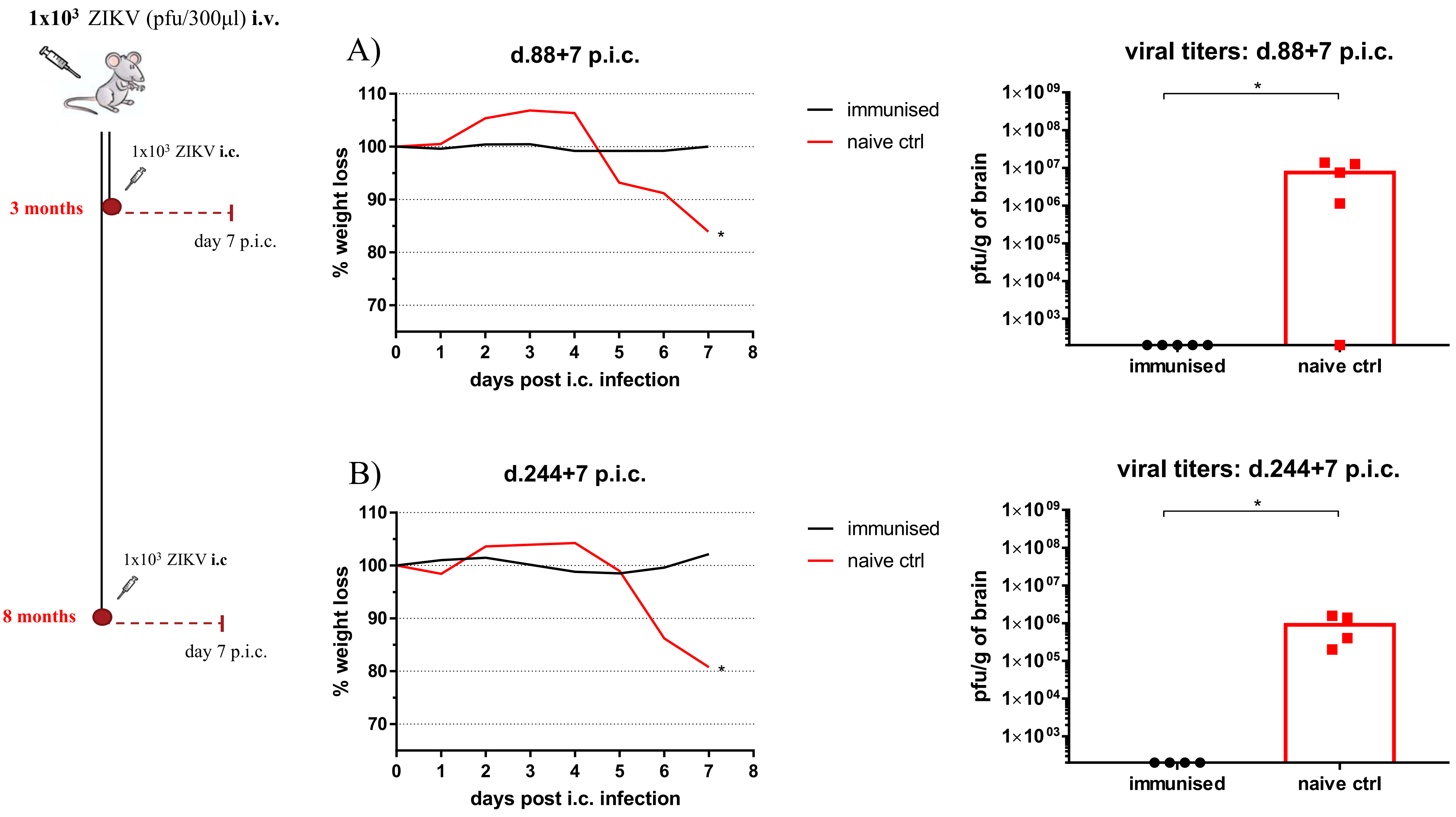

Supplementary figure 3: Longevity of protection in WT BALB/c mice. WT BALB/c mice were inoculated with 1x10^3 pfu ZIKV i.v. and 3 (A) or 8 (B) months post i.v. inoculation mice were challenged with 1x10^3 pfu ZIKV i.c. Mice were weighed and monitored daily and on day 7 post i.c. challenge brains were removed and viral titers were measured by a plaque assay. Groups of naïve mice were used as negative controls. The detection limit for virus in the brain was 250 pfu/g organ. Each dot represents an individual animal and bars the medians of the groups. The weight curves depict the group medians. *$p < 0.05$

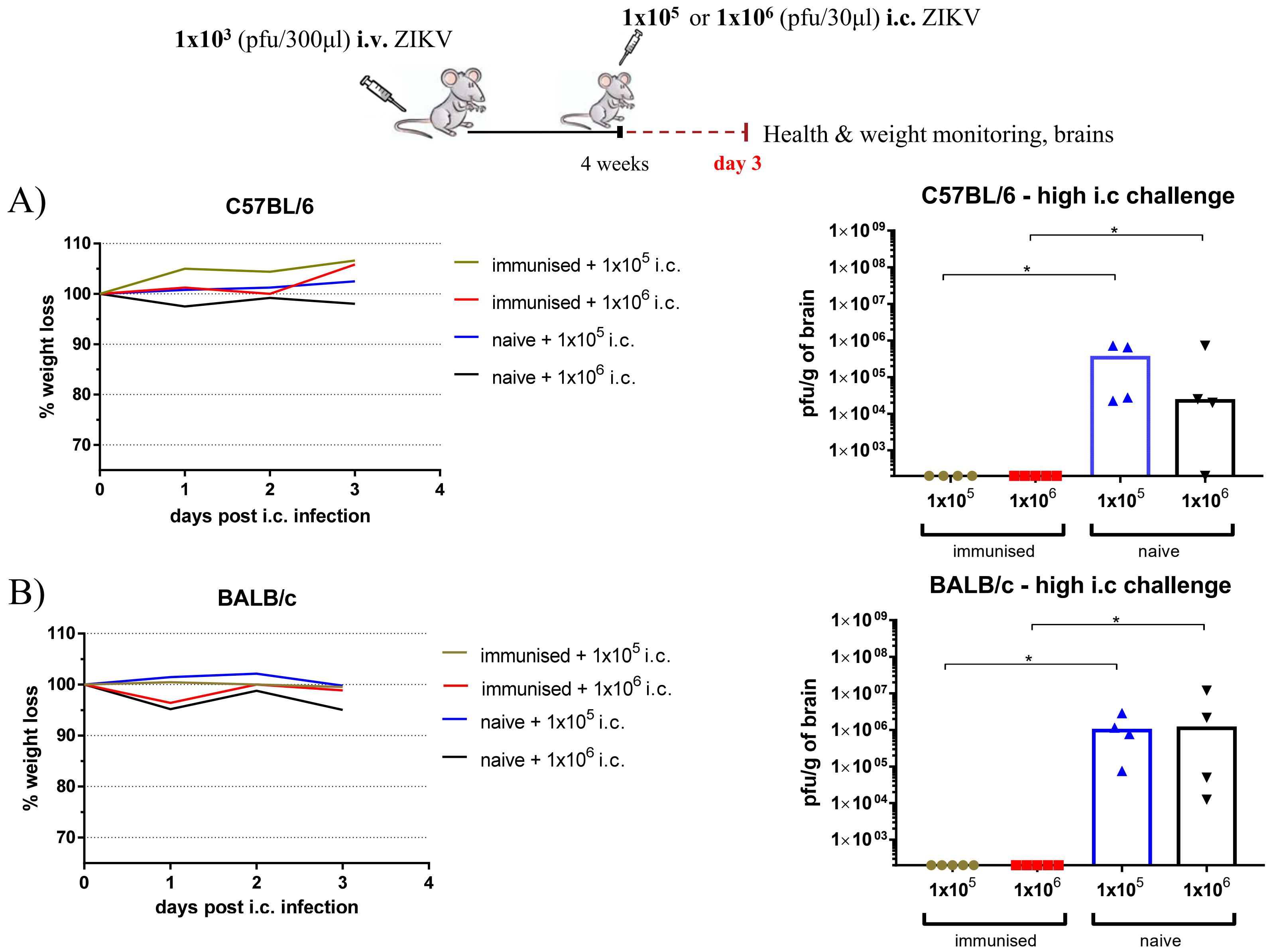

Supplementary figure 4: Increasing the i.c. challenge dose does not breach early protection. WT C57BL/6 (A) and BALB/c (B) mice were inoculated with $1x10^3$ pfu ZIKV i.v. and 4 weeks later the mice were challenged with either $1x10^5$ or $1x10^6$ pfu ZIKV i.c. Mice were weighed and monitored daily and on day 3 post i.c. challenge brains were removed and viral titers were measured by a plaque assay. Groups of naïve mice were used as negative controls. The detection limit for virus in the brain was 250 pfu/g organ. Each dot represents an individual animal and bars the medians of the groups. The weight curves depict the group medians. *$p< 0.05$.

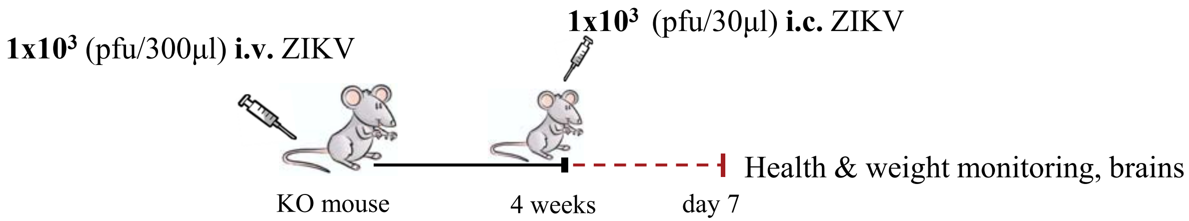

KO mouse strain	Outcome after i.c.	Conclusion
**TCRβ**<br>lack αβ TCR	Not protected	T cells are important
**myMT**<br>lack B cells	Variable protection	B cells are important
**β2 microglobulin -/-**<br>lack CD8 T cells	Protected	CD8 T cells are not essential
**CD8 -/-**<br>lack CD8 T cells	Protected	CD8 T cells are not essential
**MHC class II-/-**<br>lack CD4 T cells	Not protected	CD4 T cells are essential
**CXCR5-/-**<br>impaired germinal center formation	Semi-protected	Germinal centers are important but not crucial
**CD40L-/-**<br>impaired CD4 T-cell help	Not protected	CD4 T-cell help is essential
**IFN-γ/Prf**<br>Impaired functionality of effector T cells	Protected	Effector T cells are not essential

Supplementary table 1: Overview of the knock-out mouse strains used in this study and the level of protection observed.
